# Characterization of Naturally Acquired Immunity to a Panel of Antigens Expressed in Mature *P. falciparum* Gametocytes

**DOI:** 10.3389/fcimb.2021.774537

**Published:** 2021-11-12

**Authors:** Michelle K. Muthui, Eizo Takashima, Brian R. Omondi, Christine Kinya, William I. Muasya, Hikaru Nagaoka, Kennedy W. Mwai, Benedict Orindi, Juliana Wambua, Teun Bousema, Chris Drakeley, Andrew M. Blagborough, Kevin Marsh, Philip Bejon, Melissa C. Kapulu

**Affiliations:** ^1^ Department of Biosciences, Kenya Medical Research Institute (KEMRI)-Wellcome Trust Programme, Kilifi, Kenya; ^2^ Division of Malaria Research, Proteo-Science Center, Ehime University, Matsuyama, Japan; ^3^ School of Public Health, Faculty of Health Sciences, University of the Witwatersrand, Johannesburg, South Africa; ^4^ Department of Medical Microbiology, Radboud University Medical Centre, Nijmegen, Netherlands; ^5^ Department of Infection Biology, London School of Hygiene and Tropical Medicine, London, United Kingdom; ^6^ Division of Microbiology and Parasitology, Department of Pathology, Cambridge University, Tennis Court Road, Cambridge, United Kingdom; ^7^ Centre for Tropical Medicine and Global Health, Nuffield Department of Medicine, University of Oxford, Oxford, United Kingdom

**Keywords:** *Plasmodium falciparum*, naturally acquired immunity, mature gametocytes, seroepidemiology, malaria transmission

## Abstract

**Introduction:**

Naturally acquired immune responses against antigens expressed on the surface of mature gametocytes develop in individuals living in malaria-endemic areas. Evidence suggests that such anti-gametocyte immunity can block the development of the parasite in the mosquito, thus playing a role in interrupting transmission. A better comprehension of naturally acquired immunity to these gametocyte antigens can aid the development of transmission-blocking vaccines and improve our understanding of the human infectious reservoir.

**Methods:**

Antigens expressed on the surface of mature gametocytes that had not previously been widely studied for evidence of naturally acquired immunity were identified for protein expression alongside Pfs230-C using either the mammalian HEK293E or the wheat germ cell-free expression systems. Where there was sequence variation in the candidate antigens (3D7 *vs* a clinical isolate PfKE04), both variants were expressed. ELISA was used to assess antibody responses against these antigens, as well as against crude stage V gametocyte extract (GE) and AMA1 using archived plasma samples from individuals recruited to participate in malaria cohort studies. We analyzed antibody levels (estimated from optical density units using a standardized ELISA) and seroprevalence (defined as antibody levels greater than three standard deviations above the mean levels of a pool of malaria naïve sera). We described the dynamics of antibody responses to these antigens by identifying factors predictive of antibody levels using linear regression models.

**Results:**

Of the 25 antigens selected, seven antigens were produced successfully as recombinant proteins, with one variant antigen, giving a total of eight proteins for evaluation. Antibodies to the candidate antigens were detectable in the study population (N = 216), with seroprevalence ranging from 37.0% (95% CI: 30.6%, 43.9%) for PSOP1 to 77.8% (95% CI: 71.6%, 83.1%) for G377 (3D7 variant). Responses to AMA1 and GE were more prevalent than those to the gametocyte proteins at 87.9% (95% CI: 82.8%, 91.9%) and 88.3% (95% CI: 83.1%, 92.4%), respectively. Additionally, both antibody levels and breadth of antibody responses were associated with age and concurrent parasitaemia.

**Conclusion:**

Age and concurrent parasitaemia remain important determinants of naturally acquired immunity to gametocyte antigens. Furthermore, we identify novel candidates for transmission-blocking activity evaluation.

## 1 Introduction

Early investigations into immune responses to gametocyte antigens demonstrated that they are readily detectable in the sera of malaria exposed individuals and can sometimes develop rapidly after primary infection (Mendis et al., 1987; Graves et al., 1988; Ong et al., 1990; Bousema et al., 2006). The destruction of circulating gametocytes within the human host before transmission to mosquitoes results in gametocyte proteins being presented to the vertebrate immune system, thus stimulating an immune response (Pradel, 2007; Stone et al., 2016). The naturally acquired anti-gametocyte immune response is predominantly humoral, and work has shown that it can impact parasite development within the mosquito (Oueadraogo et al., 2018; Stone et al., 2018). Therefore, there is the potential for these immune responses to subsequently influence transmission with impact on the infectious reservoir (Stone et al., 2018). Data on naturally acquired transmission-blocking immunity may therefore inform the development of transmission-blocking vaccines (Bousema et al., 2010).

In seroepidemiological studies, serological status is defined by the presence or levels of antibodies to key parasite antigens and is used as a marker of individual or population-level parasite exposure (Polley et al., 2004; Drakeley et al., 2005; Wong et al., 2014; Kangoye et al., 2016; Idris et al., 2017). Such studies can be used to identify factors associated with the carriage of antibodies to *Plasmodium falciparum* antigens. Some of the key indicators of parasite exposure include age, location of residence and asymptomatic parasitaemia, which are commonly assessed for associations with immune responses to parasite antigens. From the seroepidemiological studies carried out so far on *P. falciparum* gametocyte antigens, based primarily on Pfs230-C and Pfs48/45, there exist discrepancies in the associations observed with age, transmission intensity and transmission season (Muthui et al., 2019a). Further work is therefore required to clarify these associations.

Several parameters influence gametocyte carriage, for example, host genetics, in particular the haemoglobinopathies that confer protection against severe malaria (Williams et al., 2005; Taylor et al., 2012; Ndungu et al., 2015). Furthermore, gametocyte carriage is also likely to impact naturally acquired immune responses to gametocyte antigens. Based on this premise, we identified a set of largely uncharacterized antigens for immunoprofiling in relation to well-studied serological markers of parasite exposure as well as risk factors for gametocyte carriage. Through this analysis, we highlight important factors that modulate the anti-gametocyte antibody response (age and concurrent parasitaemia), highlight potential markers of parasite exposure as well as new candidates that can be evaluated for transmission-blocking activity.

## 2 Methods

### 2.1 Study Design, Setting and Data Collection

Samples and epidemiological data from two cohorts were used, being the Kilifi malaria longitudinal cohort [KMLC study (Muthui et al., 2019b)] and the assessment of the infectious reservoir of malaria [AFIRM study (Gonçalves et al., 2017)]. The KMLC cohort comprised three sub-cohorts of children followed up longitudinally and sampled at cross-sectional surveys to assess asymptomatic *P. falciparum* infections. The AFIRM cohort was a cross-section sampling carried out in the wet and dry seasons and comprised children and adults. A breakdown of the cohorts is provided in **Table 1**.

**Table 1:** Summary of the cohorts included in the immunoprofiling.

Cohort	Location (s)	Study Design	Period of Sample Collection	Population Sampled	Sample Size
KMLC	Ngerenya and Junju	Cross-sectional surveys	1998 - 2016	Children	272
AFIRM	Junju	Cross-sectional (seasonally spaced)	January 2014 - February 2015	Children and Adults	216

#### 2.1.1 Kilifi Malaria Longitudinal Cohort (KMLC)

The KMLC cohort study design and sampling protocol and the subset of samples used in this study have previously been described (Mwangi et al., 2005; Bejon et al., 2006; Muthui et al., 2019b; Omondi et al., 2021). Briefly, a subset of samples was selected for the analysis, including all gametocyte positive children sampled over the 1998 – 2016 follow-up period. This gave a total of 364 samples available for analysis. Controls were selected and matched on age, sex and cohort. One set of controls was asexual parasite positive but gametocyte negative by microscopy, and the other negative for both asexual parasites and gametocytes. Not all samples were available for the eventual analysis. The final sample set consisted of 66 of the gametocyte-positive, 72 of the asexual parasite positive, and 134 of the parasite negative samples, giving 272 samples. A figure providing the sample selection process is provided in **Supplementary Figure 1**. Owing to the decline in transmission in the Ngerenya sub-cohort from 2001, the sub-cohort was divided into Ngerenya-early (1998 – 2001), a period of moderate transmission and Ngerenya-late (2002 – 2016), a period of low transmission. Similar to Ngerenya-early, the Junju sub-cohort is located within a region experiencing moderate malaria transmission.

#### 2.1.2 Assessment of the Infectious Reservoir of Malaria (AFIRM)

The AFIRM study was carried out in Junju location, Kilifi, to describe the infectious reservoir in an area of moderate malaria transmission intensity. Details of this cohort have previously been published (Gonçalves et al., 2017). The study design was cross-sectional, with one survey carried out during the rainy season and one in the dry season. Though increased transmission occurs following the onset of the rainy season, parasite transmission persists all year round along the Kenyan coast, and the timing of the rains and consequent increase in malaria transmission is irregular (Mbogo et al., 1995; Mogeni et al., 2016). There is no standard definition of the start and the end of the malaria transmission season. In our study, the start and end of the dry and wet seasons were defined using monthly rainfall data collected between 2013 – 2015. Both children and adults were recruited into the study, with different participants recruited at each cross-sectional survey. Recruitment was carried out between January 2014 and April 2014 for the dry season, with additional participants recruited between January 2015 and February 2015 and for the rainy season between May and December. Participants were recruited regardless of parasite status, with the key inclusion criteria being informed consent, age greater than two years, and willingness to provide a single 5 ml venous blood sample. The main exclusion criteria were acute disease or severe chronic conditions. At recruitment, rapid diagnostic tests were performed using Carestart RDT^®^, and any individuals presenting with parasites, by microscopy, were given a full course of anti-malarial treatment. A subset of 216 samples (72 from adults, 72 from children over five years and 72 from children under five years) was randomly selected from the main AFIRM dataset for analysis. A figure providing the sample selection process is provided in **Supplementary Figure 1**.

#### 2.1.3 Ethics Approval

The cohort studies described above were approved by the Kenya Medical Research Institute Ethics Review Committee (reference numbers KEMRI/SERU/SSC2574 - AFIRM cohort, and KEMRI/SERU/CGMRC//3149 and KEMRI/SERU/SSC1131 - KMLC cohort). The research was conducted in line with the principles of the Declaration of Helsinki, which included consenting the participants in their local language before any procedure, and obtaining written consent from the participants, or in the case of children, from their parents or guardians.

#### 2.1.4 Parasite Detection

For both the KMLC and AFIRM cohorts, data on patent parasitaemia was available. The microscopy protocol has previously been described (Mwangi et al., 2005; Muthui et al., 2019b). Briefly, blood films were prepared for the participants, fixed with 100% methanol, and subsequently stained for 45 minutes with 3% Giemsa. Enumeration of parasites on thick films was estimated relative to a count of 200 white blood cells (WBCs), while a count of 500 red blood cells (RBCs) was used for thin films. Final parasitaemia was then determined against the actual blood count or by assuming a count of either 8000 WBCs per microlitre or 5 x 10^6^ RBCs per microlitre. For the AFIRM cohort, the parasite density was calculated based on an estimate of 8000 WBCs per microlitre. Gametocyte detection was done alongside the detection of asexual parasites. All microscopy readings were carried out independently by two microscopists, with any discrepancies resolved by a third microscopist. In addition to microscopy, parasites were also detected by molecular methods for the AFIRM cohort. Detection of all parasites was done by *18S* rRNA quantitative nucleic acid sequence-based amplification (QT-NASBA) and *18S* qPCR, while specific detection of female gametocytes was carried out by *Pfs25* mRNA QT-NASBA (Schneider et al., 2004; Pett et al., 2016).

### 2.2 Identification of Antigens for Study

Candidate antigens for the study were identified from a published dataset of the gametocyte proteome (Lasonder et al., 2016). From an initial list of 2,241 proteins, we shortlisted 24 proteins for further analysis. These proteins were shortlisted based on features suggestive of surface localization (signal peptides, transmembrane domains and glycosylphosphatidylinositol anchors). An additional antigen with potential association with naturally acquired transmission reducing immunity was identified from a conference abstract (Stone et al., 2015) to give a total of 25 proteins (**Supplementary Table 1**). At the time of the search, the candidate antigens were predominantly uncharacterized as targets of naturally acquired immunity to mature gametocyte antigens. Sequence variation between the reference lab strain *P. falciparum* 3D7 (genome version 3.0) and a fully sequenced Kilifi clinical isolate – PfKE04 – was also analyzed by pairwise alignment using Geneious bioinformatics software (version 11.1.2). Where variation was identified [either an insertion/deletion or non-synonymous single nucleotide polymorphism (SNP)], both variants were prioritized for construct design.

Protein production was attempted in the wheat germ cell-free expression system (WGCFS, WEPRO7240H, Cell-Free Sciences) and, where unsuccessful in the WGCFS, in the mammalian HEK293E system. Of the 25 antigens targeted for recombinant protein production, seven antigens (including one variant antigen) were successfully expressed in sufficient quantities for this analysis. These were Pfs230-C (*PF3D7_0209000*), *PF3D7_0208800*, MDV1 (*PF3D7_1216500*), G377 (*PF3D7_1250100*, 3D7 and PfKE04 variants expressed), *PF3D7_1314500*, *PF3D7_0303900* and PSOP1 (*PF3D7_0721700*)) giving a total of 7 candidate antigens (8 proteins) for immunoprofiling analysis. Notably, owing to the size of G377 protein that would be a challenge for protein production, a domain spanning amino acids 666-1146 termed ‘domain B’ was produced for analysis. This domain was identified from work by Alano et al. (1995), who were able to produce protein from this domain successfully (Alano et al., 1995). An extensive description of the protein identification and the recombinant protein production process is provided in the **Supplementary Methods**, and results from the protein production are provided in **Supplementary Figures 2–5**. Additionally, we evaluated antibody responses to apical membrane antigen-1 protein (AMA1, *PF3D7_1133400*), a highly immunogenic asexual stage antigen (Drakeley et al., 2005; Jones et al., 2015; Idris et al., 2017) widely studied in the context of seroprevalence to place our results in context, and to a crude stage V gametocyte extract (GE).

### 2.3 Enzyme-Linked Immunosorbent Assay (ELISA)

The measurement of antibody responses to the candidate antigens, AMA1 and GE, was carried out using a previously described three-day ELISA protocol (Murungi et al., 2019; Omondi et al., 2021) with a few modifications. Additionally, to determine the optimal antigen coating concentration and serum dilution to use for the assay, checkerboard ELISAs were first carried out (**Supplementary Figures 6, 7**). On day one of the assay, 100 µL of the purified recombinant protein was prepared in coating buffer (at a concentration of 1 μg/ml for the gametocyte antigens, 0.5 μg/ml for AMA1 and 1 in 250 dilution for GE) and coated onto a 96-well plate (Immulon 4 HBX, ThermoFisher Scientific). The plate was then incubated at 4°C overnight, and on the following day, the plate was washed four times with phosphate-buffered saline containing 0.05% tween-20 (Pierce™ 20X PBS Tween™ 20 Buffer, ThermoFisher Scientific). The plate was then blocked with 200 µL of blocking buffer for five hours at room temperature and subsequently washed three times with PBS/T. One hundred microlitres of test sera were then added at dilutions of 1 in 200 for the gametocyte antigens (or 1 in 100 for lowly reactive antigens - i.e., if average optical densities were below 0.15); 1 in 1000 (or 1 in 2000 for adult sera tested) for AMA1, and 1 in 500 for GE). A standardized ELISA format was used where defined concentrations of malaria immunoglobulin (MIG) were serially diluted to generate a standard curve against which antibody units were extrapolated.

The MIG was prepared from purified immunoglobulin G (98% IgG) obtained from a pool of 834 Malawian adult sera (Taylor et al., 1992). For positive controls, a characterized pool of hyper-immune sera (PHIS) was obtained from a random selection of adult residents of Junju location, Kilifi (Osier et al., 2014; Murungi et al., 2016), and a pool of sera from gametocyte positive individuals from the AFIRM cohort with high gametocytaemia were also included. Malaria naive sera from European adults (non-immune sera, NIS) were used as negative controls at the same dilution as the test serum for each respective antigen. The NIS were tested against the measles antigen to verify that they were indeed reactive (**Supplementary Figure 8**). On day three, the plate was washed three times before 100 µL of secondary antibody (polyclonal rabbit anti-human IgG-HRP, Dako, UK) was added (1: 5,000 dilution) and the plate incubated at room temperature for three hours. The plate was washed four times before adding 100 µL of o-phenylenediamine dihydrochloride (OPD) substrate (Sigma, UK). Finally, the plate was incubated at room temperature for 15 minutes to allow colour development before the reaction was stopped using 25 µL of 2 M sulphuric acid (H_2_SO_4_). Each sample’s optical density (OD) was then determined by reading the absorbance at 492 nM. All samples were run in duplicate, with replicate samples run on a separate plate. As all samples were run in duplicate in the ELISA, the mean OD and co-efficient of variation were calculated before analysis. Samples with a coefficient of variation higher than 20% were repeated.

### 2.4 Statistical Analysis

We summarised the demographic characteristics of participants for the two cohorts separately. We did this by sub-cohort (Ngerenya-early, Ngerenya-late and Junju) within the KMLC cohort and by season (wet and dry) within the AFIRM cohort. For continuous variables, we presented their means (standard deviation) or median (interquartile range, IQR) depending on the distribution. For categorical variables, we presented the percentages with comparisons carried out using the Chi-square test. We also summarised parasite density in the different cohorts by age group, sickle cell status, α–thalassaemia status, cohort and season. Comparisons between groups were performed using the Wilcoxon rank-sum test (where a categorical variable had only two groups) or Kruskal-Wallis test (for variables with more than two groups) with posthoc analysis done using the Dunn’s test with Bonferroni correction.

Next, we estimated the seroprevalence. We note that the analysis of seroprevalence was limited to the AFIRM cohort only, where sampling cut across all age groups. Antibody responses (IgG) were measured to the candidate antigens that were successfully produced as recombinant proteins. Responses were also measured to two markers of parasite exposure: Apical membrane antigen 1 (AMA1) (Drakeley et al., 2005; Jones et al., 2015; Idris et al., 2017) and gametocyte extract (GE) (Omondi et al., 2021). A four-parameter hyperbolic standard curve was generated from the titrated MIG, and the antibody concentration of each sample was then extrapolated. A cut-off was defined as the mean antibody concentration of the NIS plus three standard deviations (3SD) to determine seropositivity. Additionally, to compare the different methods of assigning seropositivity, we also calculated seropositivity using a mixed model with the distribution of OD values fitted as the sum of two Gaussian distributions (a narrow distribution of low responders and a broader distribution of high responders) (Bousema et al., 2010). We then estimated seropositivity from a cut-off defined as the mean OD of the low responders plus 3SD. These seroprevalences were compared across variant antigens and across sampling seasons by means of a Chi-square test. The Cochrane-Armitage test was used to test for a linear trend in seroprevalence when grouped by age. Additionally, we also evaluated the dependency of seropositivity to the different antigens using a correlation matrix, with correlation analyzed using Spearman’s rank correlation.

Furthermore, we examined how determinants of parasite exposure such as age, parasite prevalence, season (AFIRM cohort), transmission intensity (KMLC cohort), and haemoglobinopathies relate to the magnitude of antibody response to the candidate antigens. These covariates were chosen owing to their associations with gametocytaemia or anti-gametocyte antibody responses (Boudin et al., 1991; Lawaly et al., 2010; Grange et al., 2015; Skinner et al., 2015; Amoah et al., 2018). Factors associated with the magnitude and breadth of antibody response to the gametocyte antigens were identified using linear regression models, and we fitted both univariable and multivariable models. Cluster-robust standard errors were calculated with clustering specified to occur by participant ID, to account for instances of repeated sampling in the KMLC cohort. For the AFIRM cohort, parasitaemia and gametocytaemia were assessed by both microscopy and the highly sensitive nucleic acid sequence-based amplification; thus, it was also possible to compare the effect of patent versus sub-patent parasitaemia and gametocytaemia in a sub-analysis.

In addition to assessing the factors associated with the magnitude of anti-gametocyte responses, we also assessed the parameters associated with the recognition of a greater number of gametocyte antigens (breadth of response). For this, we considered how the pre-specified covariates related to the breadth of antibody responses to the candidate antigens using linear regression models. All analyses were performed using R version 4.0.3 (R Core Team, 2020).

## 3 Results

### 3.1 Demographic Characteristics

#### 3.1.1 Kilifi Malaria Longitudinal Cohort (KMLC) Study

**Table 2** presents the demographic characteristics of the KMLC. In all sub-cohorts, observations were predominantly from children below five years of age. The median age across all sub-cohorts was 5.22 years (IQR 4.98 years), with the proportion under five years of age being: Ngerenya-early - 64%, Ngerenya-late - 60% and Junju - 52% (*p =* 0.302, Chi-square test). Parasite prevalence, detected by microscopy, was 78% in Ngerenya-early, 60.4% in Junju and 19% in Ngerenya-late for asexual parasites and 25% in Ngerenya-early, 33% in Junju and 8% in Ngerenya-late for gametocytes as detected by microscopy. Owing to the unavailability of a majority of samples **(** **Supplementary Figure 1** **),** it was impossible to undertake the initial matching of cases and controls, which is reflected in the variable parasite prevalence in the sub-cohorts.

**Table 2:** Demographic characteristics of observations from a subset of the KMLC cohort study participants.

	Sub-cohort		
	Ngerenya		Junju
	Early	Late	
Number of observations (N)	50	126	96
Sex: number of females (%)	25 (50.0)	40 (31.7)	45 (46.9)
Number per age group (%)			
0 - 5 years	32 (64.0)	76 (60.3)	50 (52.1)
6 - 10 years	14 (28.0)	41 (32.5)	29 (30.2)
11-15 years	4 (8.0)	9 (7.1)	17 (17.7)
Temperature (°C), median (IQR)	NM*	36.9 (36.6 - 37.1)	36.5 (36.2 - 36.9)
Number of asexual parasite positive observations (%)	39 (78.0)	24 (19.0)	58 (60.4)
Number of gametocyte positive observations (%)	25 (50.0)	8 (6.3)	33 (34.4)
Number of observations with sickle genotype (%)			
AA	49 (98.0)	103 (82.4)	70 (72.9)
AS	1 (2.0)	22 (17.6)	26 (27.1)
Number of observations with α-thalassaemia genotype (%)			
Normal	18 (40.9)	39 (31.7)	31 (32.3)
Heterozygous	21 (47.7)	65 (52.8)	48 (50.0)
Homozygous	5 (11.4)	19 (15.4)	17 (17.7)
			
**Missing data**			
Sickle genotype	.	1	.
α-thalassaemia genotype	6	3	.

*NM, not measured. A dot (.) indicates that no data were missing for the particular variable, while a number indicates the number of participants for whom corresponding genotype data were not available.

The prevalence of sickle cell trait (AS) varied among the sub-cohorts (*p* =0.001, Chi-square test) with higher prevalence in Junju (27%) and Ngerenya-late (17.6%) and lowest prevalence in Ngerenya-early (2%). The markedly low prevalence of sickle cell trait in Ngerenya-early likely results from an artefact of sample availability rather than reflecting an increase in the prevalence of sickle cell trait over time. In comparison, close to half the participants were heterozygous for α–thalassaemia (Ngerenya-early 47.7%, Ngerenya-late 52.8% and Junju 50%, *p* = 0.430, Chi-square test). Note that were only a few missing values, except for temperature, which was not measured for the Ngerenya-early sub-cohort.

#### 3.1.2 The Assessment of the Infectious Reservoir of Malaria (AFIRM) Study

**Table 3** presents the demographic characteristics for AFIRM. The median age of the study participants was 10 years (IQR 21 years). Though there were slightly fewer participants sampled in the dry season (96 participants) than the wet season (120 participants), there were no statistically significant differences in the numbers of participants in each of the age groups between the two seasons. When parasitaemia was detected using rapid diagnostic tests, there was higher parasite prevalence in the wet season (28.3% *vs* 12.5% in the wet and dry seasons, respectively, *p* = 0.008, Chi-square test). Conversely, when parasite detection was by microscopy, no significant differences in parasite prevalence were observed between the wet and dry season (16.7% *vs* 8.3% (*p* = 0.108, Chi-square test) for asexual parasites and 1% *vs* 3.3% (*p* = 0.511, Chi-square test) for gametocytes, respectively). Similarly, no differences were observed when detection was by molecular methods for all parasites (*18S* qPCR: 32.5% *vs* 41.7% (*p* = 0.212) and NASBA *18S*: 49.2% *vs* 50% (*p* = 1) in the wet and dry seasons respectively, Chi-square test) and gametocytes [NASBA *Pfs25:* 24% *vs* 25.8% in the wet and dry seasons respectively (*p* = 0.874, Chi-square test)]. There were no differences between the proportion of participants with the different sickle cell and α–thalassaemia genotypes between the wet and dry seasons.

**Table 3:** Demographic characteristics of participants from the AFIRM cohort study.

	Season	
	Dry	Wet
Number of participants (N)	96	120
Sex: number of females (%)	61 (63.5)	61 (50.8)
Number per age group (%)		
0 - 5 years	27 (28.1)	45 (37.5)
6 - 15 years	33 (34.4)	39 (32.5)
>15 years	36 (37.5)	36 (30)
Temperature (°C), median (IQR)	36.6 (36.3 - 37.0)	36.6 (36.2 - 36.8)
Number RDT positive (%)	12 (12.5)	34 (28.3)
Parasite Prevalence		
qPCR (*18S*)	40 (41.7)	39 (32.5)
NASBA* (*18S*)	48 (50)	59 (49.2)
Asexual parasite prevalence - Microscopy (%)	8 (8.3)	20 (16.7)
Gametocyte prevalence (%)		
Microscopy	1 (1.0)	4 (3.3)
NASBA (*Pfs25*)	23 (24)	31 (25.8)
Sickle genotype (%)		
AA	77 (80.2)	102 (85)
AS	19 (19.8)	18 (15)
α - thalassaemia genotype (%)		
Normal	30 (31.3)	38 (31.7)
Heterozygous	51 (53.1)	56 (46.7)
Homozygous	15 (15.6)	26 (21.7)

*NASBA, nucleic acid sequence-based amplification.

### 3.2 Relationship Between Parasite Density and the Covariates

**Figure 1:**
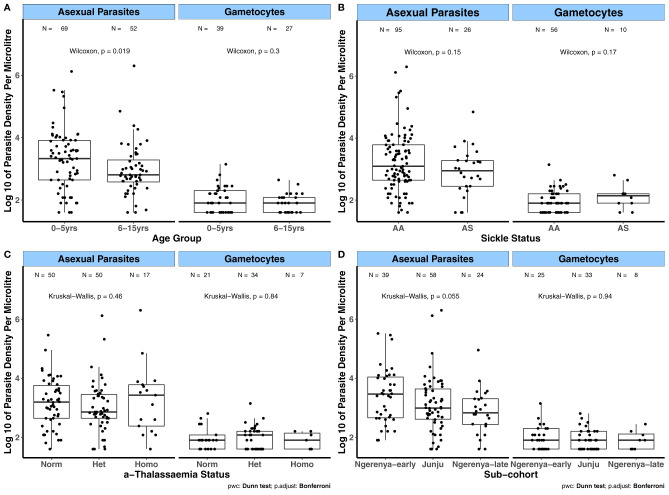
Variation in patent parasite densities by factors associated with gametocyte carriage in the KMLC cohort. Asexual parasite and gametocyte densities by **(A)** Age group; **(B)** Sickle genotype; and **(C)** α-thalassaemia genotype. **(D)** Sub-cohort (moderate transmission (Ngerenya-early and Junju) and low transmission (Ngerenya-late)). Comparisons were carried out using the Wilcoxon test and Kruskal-Wallis test (post-hoc analysis after Kruskal-Wallis carried out using Dunn’s test with Bonferroni correction). The number of parasite positive individuals (N) is indicated at the top of each graph. α-thalassaemia: Norm – normal, Het – heterozygous, and Homo – homozygous. The boxes of boxplots display the median bound by the first and third quartiles, with the whiskers depicting the lowest and highest values (excluding outliers). The dots indicate individual data points.

**Figure 2:**
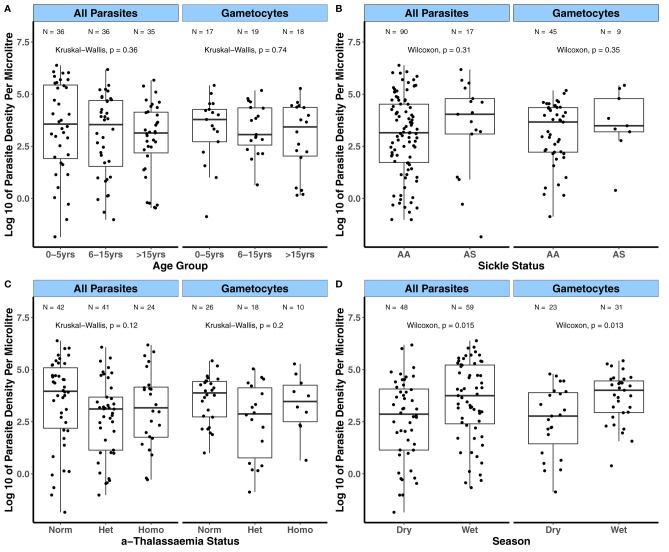
Variation in sub-patent parasite densities by factors associated with gametocyte carriage in the AFIRM cohort. Asexual parasite and gametocyte densities by **(A)** Age group; **(B)** Sickle genotype; **(C)** α-thalassaemia genotype; and **(D)** Season with parasitaemia detected by *18S* NASBA (all parasites) and *Pfs25* NASBA (female gametocytes). Comparisons were carried out using the Wilcoxon test and Kruskal-Wallis test. The number of parasite positive individuals (N) is indicated at the top of each graph. α-thalassaemia: Norm – normal, Het – heterozygous, and Homo – homozygous. The boxes of boxplots display the median bound by the first and third quartiles, with the whiskers depicting the lowest and highest values (excluding outliers). The dots indicate individual data points.

Prior to evaluating the relationships between the magnitude of antibody responses to the candidate antigens and the covariates age, transmission intensity (sub-cohorts), season and sickle cell and α-thalassaemia genotypes, we first analyzed how these covariates relate to parasite densities. This was done to evaluate whether the influence of these covariates on parasite densities relates to their impact on the magnitude of antibody responses to the candidate antigens. For the KMLC, microscopically determined asexual parasite densities were higher in children aged between 0 – 5 years of age compared to those aged 6 – 5 years (*p* = 0.019, **Figure 1A** **).** In contrast, gametocyte densities were similar across age groups. There were no differences in both asexual and gametocyte densities among the different sickle (**Figure 1B**) and α-thalassaemia genotypes (**Figure 1C**) or across the subcohorts (**Figure 1D**). As few participants had patent gametocytaemia in the AFIRM cohort (**Table 3**), we compared sub-patent parasite densities by the covariates mentioned above. In this case, sub-patent gametocyte densities did not vary by age (**Figure 2A**) or by sickle trait (**Figure 2B**) and α-thalassaemia genotype (**Figure 2C**). However, parasite densities were higher in the wet season compared to the dry season, and this was statistically significant for both all parasites (*p* = 0.015) and gametocytes (*p* = 0.013, **Figure 2D**).

### 3.3 Seroprevalence to the Candidate Antigens

For seropositivity estimated from a population of malaria naïve individuals, there was a broad range in the seroprevalence estimates, ranging from 37.0% (95% CI: 30.6%, 43.9%) for PSOP1 to 77.8% (95% CI: 71.6%, 83.1%) for G377B 3D7 variant (**Table 4**). Seroprevalence to G377B PfKE04 was similarly high (70.4%, 95% CI: 63. 8%, 76.4%), and there was a significant association between participants who responded to G377B 3D7 and those who responded to G377B PfKE04 (χ^2^ statistic = 110, *p <*0.0001; Chi-square test of independence with Yates’ continuity correction, 2 x 2 tables provided in **Supplementary Table 2**). Seroprevalence to Pfs230-C was estimated as 64.2% (95% CI: 55.9%, 71.9%), with responses to both AMA1 and GE being the most prevalent at 87.9% (95% CI: 82.8%, 91.9%) and 88.3% (95% CI: 83.1%, 92.3%) respectively.

**Table 4:** Seroprevalence of antibody responses to the gametocyte antigens, AMA1 and gametocyte extract.

Gene ID	Gene name^a^	N^b^	Median OD (Range)^C^	Seropositive	Prevalence (95% CI)
PF3D7_0209000	P230	148	0.22 (0.02, 2.50)	95	64.2 (55.9, 71.9)
PF3D7_1314500	–	216	0.35 (0.00, 3.34)	132	61.1 (54.3, 67.7)
PF3D7_0303900	–	216	0.22 (0.00, 2.81)	120	55.6 (48.7, 62.3)
PF3D7_0721700	PSOP1	216	0.24 (0.00, 2.53)	80	37.0 (30.6, 43.9)
PF3D7_0208800	–	216	0.37 (0.00, 2.49)	122	56.5 (49.6, 63.2)
PF3D7_1216500	MDV1	216	0.38 (0.00, 3.50)	126	58.3 (51.5, 65.0)
PF3D7_1250100	G377B 3D7	216	0.64 (0.00, 3.04)	168	77.8 (71.6, 83.1)
–	G377B PfKE04	216	0.59 (0.00, 2.94)	152	70.4 (63. 8, 76.4)
PF3D7_1133400	AMA1	215	1.00 (0.00, 3.68)	189	87.9 (82. 8, 91.9)
–	Gametocyte Extract	205	1.01 (0.00, 2.45)	181	88.3 (83.1, 92.4)

a Where no parasite line is specified after the gene name, the gene sequence was based on the P. falciparum reference lab isolate 3D7. PfKE04 refers to a sequenced clinical isolate obtained from a clinical sample from the KMLC.

b Number of samples assayed for each antigen; Though the total AFIRM sample set consisted of 216 individuals, not all samples were tested for Pfs230-C or GE as antigen quantities were limited.

^C^OD, optical density; SD, standard deviation. The optical density is relative to the serum dilution used, with the gametocyte antigens tested at 1:200 (except for PEB-P and PSOP1 tested at 1:100), AMA1 tested at either 1:1000 or 1:2000 and GE 1:500.

**Figure 3:**
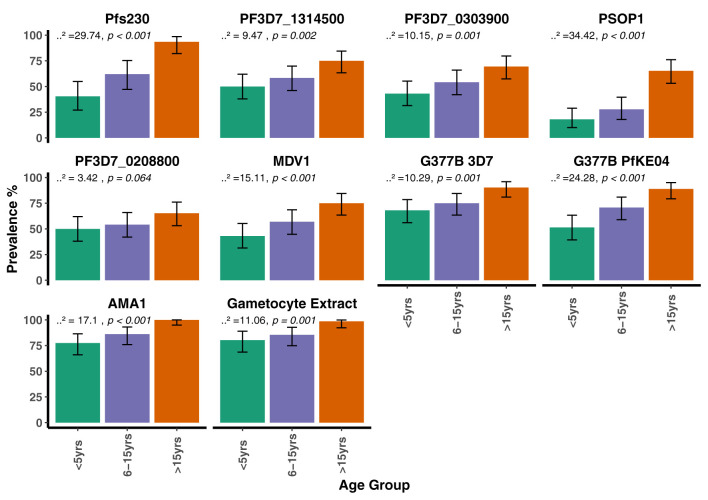
Seroprevalence to the candidate antigens, AMA1 and gametocyte extract stratified by age group in the AFIRM Cohort. Bar plots showing the prevalence of antibodies to the candidate antigens, AMA1 and gametocyte extract within the different age categories. The Cochran-Armitage test for trend was used to analyze the relationship between seroprevalence and age; *p* values are presented at the top of each panel. Error bars show 95% binomial confidence intervals (Clopper–Pearson interval).

**Figure 4:**
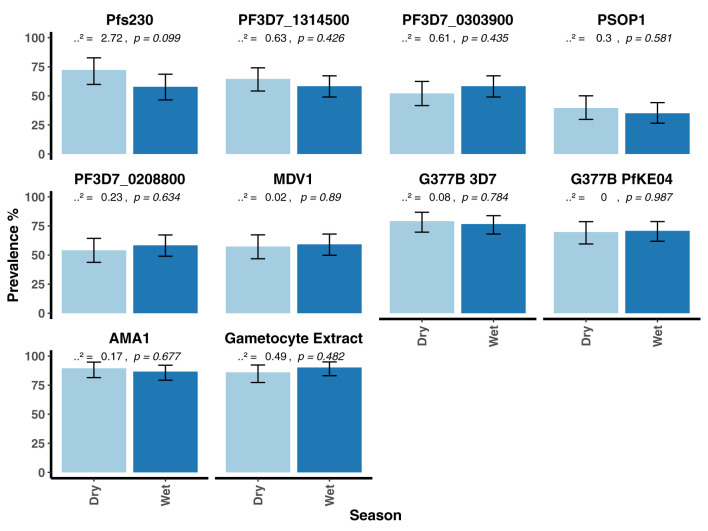
Seroprevalence to the candidate antigens, AMA1 and gametocyte extract stratified by season in the AFIRM cohort. Bar plots showing the prevalence of antibodies to the candidate antigens, AMA1 and gametocyte extract in the dry and the wet seasons. A Chi-square-test was used to compare proportions between the dry and wet seasons. Respective *p* values are presented at the top of each panel. Error bars show 95% binomial confidence intervals (Clopper–Pearson interval).

When seroprevalence was stratified by age, with age categorized as 0 – 5 years, 6 – 15 years and >15 years, there was a general trend towards increasing seroprevalence with age for all antigens being statistically significant for all except PF3D7_0208800 (*p* = 0.064, **Figure 3**). There was no evidence for a difference in seroprevalence between the dry and wet seasons for any of the antigens (**Figure 4**). When seroprevalence was estimated using a subset of low responders as the seronegative population, the estimated seroprevalence was lower, particularly for the gametocyte antigens, likely reflecting the low ODs in the seropositive samples (**Supplementary Table 3**). Despite this, the trend towards increasing seroprevalence with age remained significant for all antigens, except for PEB-P (**Supplementary Figure 9**). Seroprevalence did not associate with season same as before (**Supplementary Figure 10**).

We also examined the dependency between antibody responses to the candidate gametocyte antigens, AMA1 and GE using a correlation matrix (**Supplementary Figure 11** and **Supplementary Table 4**). The strongest correlation was observed between seropositivity to the two G377B variants (Spearman’s correlation ρ = 0.75, *p <*0.0001). Furthermore, there was a trend towards a stronger correlation between seropositivity to the different gametocyte antigens than between seropositivity to the gametocyte antigens and positivity to either AMA1 or GE. While seropositivity to AMA1 and GE did not strongly correlate with seropositivity to the gametocyte antigens, their strongest correlation was with each other (Spearman’s correlation ρ = 0.51, *p <*0.0001).

### 3.4 Factors Associated With the Magnitude of Antibody Response: KMLC Cohort

Detailed results from the univariable and multivariable analysis of predictors of antibody levels are provided in **Supplementary Tables 5, 6**, with the results of the multivariable analyses discussed below.

Statistically significant associations between increased age and increased magnitude of antibody responses to AMA1, Pfs230-C, G377B 3D7 and G377B PfKE04 were observed. Furthermore, patent asexual parasitaemia was significantly associated with an increased magnitude of antibody responses to all antigens. Gametocytaemia, on the other hand, was only significantly associated with increased responses to AMA1, GE, PF3D7_1314500, PF3D7_0303900, and PF3D7_0208800 (*p <*0.05). No significant associations were observed between sickle cell trait and antibody levels for any of the antigens. Conversely, being heterozygous (estimate -0.24, 95% CI: -0.45, -0.03, *p* = 0.022) or homozygous (estimate -0.30, 95% CI: -0.57, -0.04, *p* = 0.025) for α-thalassaemia was associated with reduced responses to GE. Generally, transmission intensity did not appear to predict the magnitude of antibody response to the gametocyte antigens independent of parasite prevalence, except in the case of GE and G377B 3D7. Relative to the Junju sub-cohort (moderate transmission intensity), residing in the low transmission sub-cohort (Ngerenya-late) was associated with reduced immune responses to GE (estimate -0.30, 95% CI: -0.54, -0.06, *p* = 0.015) and G377B 3D7 (estimate -0.12, 95% CI: -0.23, -0.004, *p* = 0.043).

Both G377B 3D7 and G377B PfKE04 had similar associations between increasing age and increased magnitude of antibody responses, and increased antibody levels with concurrent parasitaemia. Additionally, R^2^ values of the predictive models for both variants were similar (G377B 3D7 R^2^ = 0.36, G377B PfKE04 R^2^ = 0.36).

### 3.5 Factors Associated With the Magnitude of Antibody Response: AFIRM Cohort

Detailed results of the univariable and multivariable analyses are provided in **Supplementary Tables 7, 8**, with a summary of the results of the multivariable analyses provided below.

There was a statistically significant trend towards increasing magnitude of antibody responses with increasing age for Pfs230-C, AMA1 and GE, with only older age (>15 years of age) associated with an increased antibody response (*p <*0.05) to all other antigens except for PF3D7_0208800. Similar to the KMLC, parasitaemia was an important predictor of the magnitude of antibody response to the gametocyte antigens, with the exception of PSOP1. Both patent and sub-patent parasitaemia were associated with an increased magnitude of antibody responses to PF3D7_1314500, PF3D7_0303900, MDV1, G377B (both variants) and GE. To explore whether patent parasitaemia better predicted the magnitude of antibody responses to these antigens, a second analysis limited to positive parasite individuals was carried out **(** **Supplementary Tables 9, 10** **)**. There was no indication that patent parasitaemia was a better predictor of the magnitude of antibody responses relative to sub-patent parasitaemia in this sub-analysis. Moreover, in the adjusted analysis, gametocytaemia did not independently predict the magnitude of antibody responses to the gametocyte antigens except for Pfs230-C (estimate 0.23, 95% CI: 0.02 – 0.44, *p* = 0.030).

Unlike in the KMLC, where no associations with sickle cell were seen, sickle cell trait was associated with a reduced magnitude of antibody response to PF3D7_0303900 in both the univariable and multivariable analysis (multivariable analysis: estimate -0.24, 95% CI: -0.42 – 0.05, *p* = 0.013). No associations among the α-thalassemia genotypes and antibody levels were seen. Similarly, no association was observed between sampling season and antibody levels for any of the antigens.

Similar to the KMLC, both variants of G377B had associations between increasing age and increased magnitude of antibody responses and between and concurrent parasitaemia and increased level of antibody responses. Additionally, the R^2^ values of models predicting the magnitude of antibody responses to either antigen varied marginally (G377B 3D7 R^2^ = 0.34, G377B PfKE04 R^2^ = 0.36).

### 3.6 Factors Associated With the Breadth of Response

**Figure 5:**
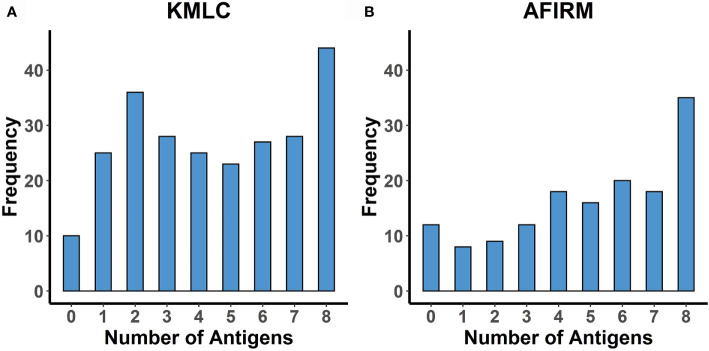
Breadth of response to the eight gametocyte proteins. Histograms showing the number of study participants seropositive to an increasing number of the proteins studied (limited to participants with responses measured to all eight proteins). **(A)** KMLC (N = 246) and **(B)** AFIRM cohort (N = 148).

The mean (standard deviation) breadth of response to the eight gametocyte proteins was 4.5 (2.5) in the KMLC cohort and 5 (2.6) in the AFIRM cohort (**Figure 5**).

Adjusted linear regression analysis of factors associated with increased breadth indicated an association between increasing age and increased antibody breadth for both cohorts (**Tables 5** and **6**). Additionally, concurrent parasitaemia, whether asexual parasites or gametocytes, was also associated with increased breadth. No statistically significant associations with sickle and α-thalassemia genotypes, transmission intensity or season were observed.

**Table 5:** Linear regression analysis of the factors influencing the number of antigens recognized by the study participants – KMLC cohort.

Covariate	Univariable			Multivariable		
	Estimate	95% CI	*p* value	Estimate	95% CI	*p* value
Age Group						
0 - 5 years	Ref.	.	.	Ref.	.	.
6 - 10 years	0.11	0.03, 0.2	**0.0091**	0.11	0.03, 0.18	**0.0044**
11 - 15 years	0.1	-0.04, 0.25	0.1692	0.08	-0.06, 0.21	0.2528
Asexual parasite positive	0.29	0.22, 0.37	**<0.001**	0.23	0.14, 0.33	**<0.001**
Gametocyte positive	0.23	0.15, 0.3	**<0.001**	0.12	0.02, 0.22	**0.0217**
Sickle						
Normal	Ref.	.	.	Ref.	.	.
Heterozygous	0.04	-0.06, 0.15	0.4201	0	-0.09, 0.09	0.9724
α - thalassaemia						
Normal	Ref.	.	.	Ref.	.	.
Heterozygous	-0.04	-0.13, 0.05	0.3870	-0.01	-0.09, 0.07	0.8805
Homozygous	-0.03	-0.15, 0.09	0.6393	0.01	-0.1, 0.11	0.8794
Cohort						
Junju	Ref.	.	.	Ref.	.	.
Ngerenya-early	0.05	-0.11, 0.22	0.5226	-0.01	-0.13, 0.11	0.8459
Ngerenya-late	-0.17	-0.28, -0.05	**0.0048**	-0.06	-0.15, 0.03	0.2008

a Parasitaemia as determined by microscopy.

Ref., reference category. P values in bold are statistically significant (p<0.05).

**Table 6:** Linear regression analysis of the factors influencing the number of antigens recognized by the study participants – AFIRM cohort.

Covariate	Univariable			Multivariable		
	Estimate	95% CI	*p* value	Estimate	95% CI	*p* value
Age Group						
0 - 5 years	Ref.	.	.	Ref.	.	.
6 - 10 years	0.12	0.02, 0.21	**0.0192**	0.11	0.02, 0.20	**0.0160**
11 - 15 years	0.31	0.21, 0.41	**<0.001**	0.31	0.22, 0.40	**<0.001**
Asexual parasite positive	0.23	0.14, 0.31	**<0.001**	0.18	0.09, 0.26	**<0.001**
Gametocyte positive	0.21	0.11, 0.31	**<0.001**	0.10	-0.002, 0.20	**0.0578**
Sickle						
Normal	Ref.	.	.	Ref.	.	.
Heterozygous	-0.06	-0.18, 0.05	0.2925	-0.06	-0.15, 0.04	0.2593
α - thalassaemia						
Normal	Ref.	.	.	Ref.	.	.
Heterozygous	-0.05	-0.14, 0.05	0.3634	0.004	-0.08, 0.09	0.9297
Homozygous	0.06	-0.06, 0.19	0.3303	0.08	-0.03, 0.19	0.1386
Season						
Dry	Ref.	.	.	Ref.	.	.
Wet	-0.01	-0.1, 0.07	0.7701	0.004	-0.07, 0.08	0.8964

a Parasitaemia as determined by PCR (18S QT-NASBA for all parasites and Pfs25 mRNA QT-NASBA for female gametocytes).

Ref., reference category. P values in bold are statistically significant (p<0.05).

## 4 Discussion

We carried out seroepidemiological analyses on a set of relatively uncharacterized mature stage V gametocyte proteins, using sera from two cohorts of malaria-exposed individuals. The aim of this work was to improve our understanding of naturally acquired immunity to sexual stage antigens that had previously been poorly characterized and hence identify new targets of naturally acquired immunity other than Pfs230-C and Pfs48/45. This is the first study of naturally acquired antibody responses in Kenya to use a panel of gametocyte antigens, as well as the first to describe patterns of seroprevalence to PF3D7_02080800.

Key determinants of malaria exposure as well as risk factors for gametocyte carriage were used to describe the dynamics of antibody responses to the candidate antigens. To place our results in the context of what is known, our list of candidates included the widely studied gametocyte/gamete antigen Pfs230-C (Bousema et al., 2007; Jones et al., 2015; Amoah et al., 2018; Oueadraogo et al., 2018; Stone et al., 2018) as well as two markers of parasite exposure AMA1 (Polley et al., 2004; Drakeley et al., 2005; Wong et al., 2014; Kangoye et al., 2016; Idris et al., 2017) and a crude extract prepared from a culture of mature gametocytes (GE) (Omondi et al., 2021). In this way, our study provides a more extensive profiling of the naturally acquired anti-gametocyte response than has been done in previous studies.

Antibodies to the panel of individual antigens tested were readily detected in the two cohorts analysed indicating that they are likely targets of naturally acquired immunity to gametocytes. Further investigations into their transmission-blocking activity are warranted, particularly for PF3D7_1314500, PF3D7_0303900 and PF3D7_0208800 that have not yet been tested as transmission-blocking vaccine (TBV) candidates, and for G377B that has similar patterns of association with Pfs230-C. Seroprevalence to the gametocyte antigens was quite varied. The highest seroprevalence was observed for G377B (both variants), with seroprevalence to Pfs230-C estimated as 64.2%. Lowest seroprevalence was seen for PSOP1. Though peak expression of PSOP1 may be observed at the ookinete stage, low-level expression may begin at the gametocyte stage as has been observed for its *P. berghei* homologue (Khan et al., 2005). Estimates of seroprevalence to Pfs230 from previous studies have varied widely, with variation attributable to differences in study design and immunoassay protocol such as 1) antigen coating concentration, 2) serum dilution, and 3) method used to determine the seropositivity cut-off (Muthui et al., 2019a). Therefore, seroprevalence estimates from different studies may not be directly comparable unless study designs and assay methodologies are similar, and this makes a case for standardized protocols for carrying out such studies. We found evidence for an age-dependent increase in seroprevalence to the candidate antigens. This may support the argument that malaria-exposed individuals develop some degree of long-lived antibody responses to sexual stage antigens with age (cumulative exposure). This may be important for natural boosting of TBV-induced antibody responses in the field, thereby making the TBV more efficacious.

Increased seroprevalence in the wet season has also been described (Ouédraogo et al., 2011; Skinner et al., 2015; Oueadraogo et al., 2018). However, it is important to note that these studies were done in areas with a pronounced difference in parasite prevalence between the dry and wet seasons, unlike the areas sampled in our study, and hence this may explain why we did not find evidence for an impact of season on seroprevalence.

In addition to seroprevalence, we also examined factors that may influence the magnitude of antibody responses to gametocyte antigens using data from two cohorts in separate analyses conducted using linear regression models. As with seroprevalence, there was evidence to suggest an influence of age on antibody levels, with the strongest evidence seen for Pfs230-C and G377B (both variants). In contrast, there was no evidence for an age-dependent increase in the magnitude of antibody responses to PF3D7_0208800. For PF3D7_0208800, concurrent parasitaemia was a better predictor of the magnitude of antibody responses rather than age. Concurrent parasitaemia was a strong predictor of the magnitude of antibody responses to all the candidate gametocyte antigens as well as to AMA1 and GE. Asexual parasitaemia is a crucial determinant of gametocyte carriage (Bousema et al., 2004; Ouédraogo et al., 2011; Koepfli et al., 2014) and hence can influence antibodies to gametocyte antigens. In addition to influencing the magnitude of antibody response, both age and concurrent parasitaemia were associated with an increased breadth of response to the gametocyte antigens. These findings are in line with what was reported by Osier et al. (2008) for a panel of asexual stage antigens.

Some studies have shown that haemoglobinopathies may increase gametocyte carriage (Trager and Gill, 1992; Lawaly et al., 2010). We hypothesized that this might correlate with an increase in the magnitude of antibody responses to the candidate antigens. However, we found that there was no clear evidence to suggest associations between either of the sickle or α-thalassaemia genotypes and the magnitude of antibody responses against the gametocyte antigens, as any trends observed were inconsistent across the two cohorts. A clearer understanding of the potential role of haemoglobinopathies in modulating responses to sexual stage antigens may be better discerned using longitudinal cohorts with frequent sampling over time.

For G377, variants based on 3D7 and a clinical isolate (PfKE04) were tested. Polymorphisms in malaria vaccine candidate antigens reduce their efficacy in the field (Genton et al., 2002; Laurens et al., 2013; Lyke, 2017; Draper et al., 2018); therefore, this is an essential factor to consider. Gametocyte antigens are thought to be relatively conserved (Doumbo et al., 2018). However, sequence variation does exist for some antigens. Acquah et al. (2017) investigated the impact of variation in the 6C region of Pfs48/45 (a single non-synonymous SNP present in 63.2% of the tested samples) and C0 region of Pfs230-C (nine base pair deletion present in 31% of the tested samples) on antibody responses. They found no impact of harbouring parasites with these variations on antibody responses to either antigen. In our study, seroprevalence did not differ between variants of G377B. Additionally, associations between either increasing age or concurrent parasitaemia and increased magnitude of antibody response were observed for both G377B variants. This could point to variant-transcendent responses to the G377B variants we evaluated; however, further experiments will be required to confirm this.

While the two cohorts allowed independent evaluation of some of the factors relating to malaria exposure to predict responses to the gametocyte antigens, differences in study design and variables evaluated did not allow pooling of the studies; for instance, data on submicroscopic parasite carriage was not available in the KMLC cohort. Additionally, the cross-sectional nature of these cohorts did not allow discerning the relationship between recent versus prior parasite exposure and sexual stage antibody kinetics (Jones et al., 2015; Oueadraogo et al., 2018). Future longitudinal studies, including data on patent and sub-patent parasite densities (Ouédraogo et al., 2011) as well as infectivity at several time points (Stone et al., 2018), would be key to better defining both the kinetics of sexual stage immunity and its impact on infectivity. Moreover, such studies may need to incorporate a range of transmission intensities (from high to low transmission settings) to correctly define the impact of parasite exposure. Lastly, we analyzed a small panel of antigens which we endeavoured to produce as full-length ectodomains where possible. However, while we were able to identify previously defined patterns of association, a much larger panel of antigens may be required to wholly elucidate naturally acquired sexual stage immunity.

In summary, the key determinants of antibody responses to the gametocyte proteins explored in this analysis were concurrent parasitaemia and age. Notably, the strong association between age and antibody responses to Pfs230-C and G377B may serve as evidence for an age-dependent acquisition of antibody responses to some gametocyte antigens as seen with asexual stage antigens. However, whether this translates to functional immunological memory requires further investigation. Additionally, the ability of sub-patent parasitaemia to boost antibody levels highlights the importance of considering chronic infections when describing the dynamics of naturally acquired responses to gametocyte antigens. PF3D7_0303900, PF3D7_1314500, PF3D7_0208800 and GE appeared to have potential as serological markers of high-density gametocytaemia. This was especially true for PF3D7_0208800, where concurrent parasitaemia, rather than age, predicted the magnitude of response. The evidence presented here warrants further evaluation of their prognostic ability in longitudinal cohort studies as they may provide valuable tools for assessing the infectious reservoir. Furthermore, these antigens may have utility as indicators of populations where TBV implementation should be prioritised, or be used to monitor the success of TBV implementation.

## Data Availability Statement

The datasets presented in this study can be found in online repositories. The names of the repository and accession number can be found here: Harvard Dataverse online repository through the link https://doi.org/10.7910/DVN/WL8TRW.

## Ethics Statement

The studies involving human participants were reviewed and approved by the Kenya Medical Research Institute Ethics Review Committee (reference numbers KEMRI/SERU/SSC2574 - AFIRM cohort, and KEMRI/SERU/CGMRC//3149 and KEMRI/SERU/SSC1131 - KMLC cohort). Written informed consent to participate in this study was provided by the participants’ legal guardian/next of kin.

## Author Contributions

MM and MK conceived and designed the study. MM, ET, BRO, CK, WM, and HN performed the experiments. MM, KM, and BO performed the statistical analysis. MM wrote the paper. JW, TB, CD, AB, KM, MK, and PB set up the cohorts of participants and participated in the drafting of the manuscript. All authors contributed to the preparation of the article and approved the submitted version.

## Funding

The AFIRM study was supported by a Bill and Melinda Gates Foundation grant (OPP1034789) to CD and TB. The KMLC study was supported by a grant from the Wellcome Trust (092741). MM, BRO, CK, and WM are IDeAL scholars whose studentships are funded by DELTAS Africa Programme *via* the Wellcome Trust grant (107769) to KEMRI-Wellcome Trust Research Programme. ET is funded by a JSPS KAKENHI grant (JP21H02724). PB and MK were supported by a Wellcome Trust grant (107499). AMB thanks the MRC (MR/N00227X/1), Isaac Newton Trust, Alborada Fund, Wellcome Trust ISSF and University of Cambridge JRG Scheme, GHIT, Rosetrees Trust and the Royal Society for funding.

## Conflict of Interest

The authors declare that the research was conducted in the absence of any commercial or financial relationships that could be construed as a potential conflict of interest.

## Publisher’s Note

All claims expressed in this article are solely those of the authors and do not necessarily represent those of their affiliated organizations, or those of the publisher, the editors and the reviewers. Any product that may be evaluated in this article, or claim that may be made by its manufacturer, is not guaranteed or endorsed by the publisher.
