# Supplementary material for: Characterization of Naturally Acquired Immunity to a Panel of Antigens Expressed in Mature P. falciparum Gametocytes
Source: Front Cell Infect Microbiol. 2021 Nov 12;11:774537. doi: 10.3389/fcimb.2021.774537 (PMC8633105; doi:10.3389/fcimb.2021.774537)
Supplement: Supplementary file 1 [file DataSheet_1.docx]

Supplementary Material

# Supplementary Data

## Supplementary Methods

### Identification of antigens for study

Candidate antigens for the study were identified from a published dataset of the gametocyte proteome (Lasonder et al., 2016). From a dataset of 2,241 proteins identified in a mixed culture of *P. falciparum* male and female gametocytes, we shortlisted 430 antigens by enriching the published dataset for proteins highly expressed in stage V gametocytes. Predicted intracellular proteins (ribosomal proteins, intracellular enzymes and transcription factors) were then filtered out. The remaining list of 97 proteins was combined with a list of 16 previously identified potential molecular biomarkers of gametocytaemia (Kapulu M, personal communication). These biomarkers were downselected from a previously identified list using mRNA expression data from published datasets (Silvestrini et al., 2005, 2010; Young et al., 2005; Lemieux et al., 2009; Eksi et al., 2012; Ikadai et al., 2013). The gene IDs corresponding to the candidate antigens were searched on PlasmoDB Release 31, 9th March 2017, (Aurrecoechea et al., 2009) and the rodent malaria genetically modified parasites database (RMgmDB, <https://www.pberghei.eu/index.php>) (Khan et al., 2013). All proteins with a signal peptide were shortlisted for protein production, giving 24 potential antigens (including Pfs230 and Pfs48/45) for analysis. An additional antigen with a possible association with naturally acquired transmission reducing immunity was identified from a conference abstract (Stone et al., 2015) to give a total of 25 proteins. At the time of the search, the candidate antigens were predominantly uncharacterized as targets of naturally acquired immunity to mature gametocyte antigens. A summary of the selection process with the number of proteins identified in each step is provided in **Supplementary Figure 12**.

### Protein production and preparation of gametocyte extract

Respective DNA and protein sequences corresponding to the candidate antigens were obtained from PlasmoDB database (Release 31, 9th March 2017) using *P. falciparum* 3D7 lab strain as the reference genome (version 3.0). Additionally, we obtained sequences from a fully sequenced field isolate – PfKE04. We then analyzed sequence variation between the two isolates by pairwise alignment using Geneious bioinformatics software (version 11.1.2). Where variation was identified (either an insertion/deletion or non-synonymous single nucleotide polymorphism (SNP)), both variants were prioritized for construct design. Where possible, we aimed to produce full-length ectodomains of the target proteins. Therefore, we excluded predicted signal peptide sequences, transmembrane domains occurring at the termini of proteins and GPI anchor coding regions when designing the constructs for cloning.

### Wheat germ cell-free system protein expression and purification

Respective genes were amplified from parasite cDNA using primers designed to contain the Xho1 restriction site (or Kpn1 if an internal Xho1 restriction site was present) and a start codon in the forward primer and a Not1 restriction site and a stop codon in the reverse primer (**Supplementary Table 11**). The inclusion of these restriction sites allowed cloning into the WGCFS expression vector, pEU-E01-MCS, multiple cloning site (MCS). The generated plasmids were sequenced to confirm that the cloned product corresponded to the target sequences before protein expression. Protein expression was carried out using the WEPRO^®^ 7240H kit (CFS Co., Ltd., Matsuyama, Japan) using the bilayer translation reaction method described previously (Tsuboi et al., 2010). Briefly, transcription reactions were set up containing 25 ng of plasmid DNA, 25 mM dNTP mix, translation buffer, and 1 U/μl each of RNase inhibitor and SP6 RNA polymerase. The reaction was then incubated at 37°C for 6 hours, after which an aliquot of the mRNA was run on a 1% agarose gel to check for mRNA degradation. After confirming mRNA integrity, translation reactions were set up containing the mRNA, 40 ng/μl of creatine kinase and 120 OD/ml of wheat germ extract. The translation reaction was then incubated at 17°C for 20 hrs, and the translation mix was transferred to 4°C immediately afterwards, awaiting protein purification.

Proteins were purified by gravity flow using polypropylene columns (Qiagen) containing an appropriate volume of nickel resin (GE Healthcare Ni Sepharose® High-Performance affinity resin). Impurities were washed off using a wash buffer containing 20 mM sodium phosphate, 300 mM sodium chloride and 50 mM imidazole at pH 7.4. Proteins were then eluted from the columns using 20 mM Sodium phosphate, 300 mM sodium chloride and 500mM imidazole at pH 7.4. As the proteins were histidine-tagged, protein expression was confirmed using western blot analysis using an HRP-conjugated anti-histidine antibody (ThermoFisher Scientific, 6x-His Tag Monoclonal Antibody (3D5), HRP).

### Mammalian system protein expression and purification

Where protein expression was unsuccessful in the wheat germ system, constructs were designed for expression in the mammalian system. To prepare the constructs, endogenous signal peptide sequences were replaced with the human tissue plasminogen activator (tPA) signal sequence that has been described to enhance heterologous protein expression in eukaryotic expression systems (Golden et al., 1998; Malin et al., 1998; Biswas et al., 2011). Additionally, potential N-glycosylation sites were modified by substituting asparagine residues to glutamine residues in predicted glycosylation sequons (Asn-X-Thr/Ser to Gln-X-Thr/Ser). A Kozak consensus sequence (5’-CCACC-3’) was added to the 5’ end of the tPA signal sequence upstream of the ATG initiation codon site for enhanced translation (Kozak, 1987), and a stop codon was added at the end. The sequences were then optimized for codon usage in mammalian cells by GeneArt® (Life Technologies, Germany). In-Fusion® cloning primers compatible with cloning into the pOPINGS vector (Addgene plasmid # 41121) were designed to amplify codon-optimized gene constructs from the GeneArt®-provided vectors (**Supplementary Table 11**). The PCR products were purified by gel extraction and ligated into the pOPINGS vector for protein expression.

The Human Embryonic Kidney 293 cell line modified with the Epstein Barr virus nuclear antigen 1 (HEK293E) was used for mammalian expression. First, the HEK293E cells were grown to a density of 1.1 x 10^6^ cells/ml with minimum viability of 97%. The cells were then transfected using Lipofectamine® 3000 reagent (ThermoFisher Scientific). For a 20 ml transfection volume, 30 μg of plasmid DNA was added to 2 ml of Opti-MEM™ reduced serum media (ThermoFisher Scientific) together with 2x the volume of DNA of P3000® reagent (ThermoFisher Scientific). This mixture was then vortexed mildly and set aside. In a second tube, 2 ml of Opti-MEM™ and 30 μl of lipofectamine were added, and the combination vortexed mildly before incubation at room temperature for 5 minutes. The Opti-MEM™/lipofectamine mix was then combined with the Opti-MEM™/DNA mix and incubated at room temperature for 20 minutes. This transfection mix was then added to the HEK293E cells, and the cells were transferred to an incubator set at 37°C with 5% CO_2_ supply with shaking set at 130 rpm. Twenty-four hours post-transfection, a casein peptone mix, TN1, enriched with vitamins and growth factor, was added to a final concentration of 0.5%. The cells were left to grow for three days before the supernatant was harvested for protein purification, carried out as described for the WGCFS expressed proteins.

### Preparation of gametocyte extract (GE) and AMA1

Crude extract prepared from mature gametocytes was prepared for analysis as described (Omondi et al., 2021). Briefly, *P. falciparum* NF54 asexual parasites were cultured in complete culture media (5.96g/l HEPES, 1.96g/l glucose, 200mM L-glutamine, 50mg/l hypoxanthine and 40mg/l gentamicin and 10.4g RPMI 1640 and 10% human serum) in an incubator at conditions of 92% N2, 3% O2, 5% CO2 and temperature of 37ºC. The culture was synchronized twice by sorbitol treatment prior to gametocyte induction (at 0 – 6 hours post-invasion and 18 – 24 hours). On the induction day, the parasitaemia was diluted to 1% and adjusted to 5% haematocrit using fresh O+ RBCs. After induction, daily media changes and monitoring of gametocytaemia were carried out. Gametocyte-infected red blood cells were harvested between stages IV and V, and the cultures were pelleted before lysis using a combination of freeze-thawing and sonication. The AMA1 protein used was provided by Dr James Tuju and was expressed in the mammalian HEK293E system.

### References

Aurrecoechea, C., Brestelli, J., Brunk, B. P., Dommer, J., Fischer, S., Gajria, B., et al. (2009). PlasmoDB: A functional genomic database for malaria parasites. *Nucleic Acids Res.* 37, 539–543. doi:10.1093/nar/gkn814.

Biswas, S., Dicks, M. D. J., Long, C. A., Remarque, E. J., Siani, L., Colloca, S., et al. (2011). Transgene optimization, immunogenicity and in vitro efficacy of viral vectored vaccines expressing two alleles of plasmodium falciparum AMA1. *PLoS One* 6. doi:10.1371/journal.pone.0020977.

Eksi, S., Morahan, B. J., Haile, Y., Furuya, T., Jiang, H., Ali, O., et al. (2012). Plasmodium falciparum Gametocyte Development 1 (Pfgdv1) and Gametocytogenesis Early Gene Identification and Commitment to Sexual Development. *PLoS Pathog.* 8. doi:10.1371/journal.ppat.1002964.

Golden, A., Austen, D. A., Van Schravendijk, M. R., Sullivan, B. J., Kawasaki, E. S., and Osburne, M. S. (1998). Effect of promoters and signal sequences on the production of secreted HIV-1 gp120 protein in the baculovirus system. *Protein Expr. Purif.* 14, 8–12. doi:10.1006/prep.1998.0926.

Ikadai, H., Shaw Saliba, K., Kanzok, S. M., McLean, K. J., Tanaka, T. Q., Cao, J., et al. (2013). Transposon mutagenesis identifies genes essential for Plasmodium falciparum gametocytogenesis. *Proc. Natl. Acad. Sci. U. S. A.* 110, E1676-84. doi:10.1073/pnas.1217712110.

Khan, S. M., Kroeze, H., Franke-Fayard, B., and Janse, C. J. (2013). Standardization in generating and reporting genetically modified rodent malaria parasites: the RMgmDB database. *Methods Mol. Biol.* 923, 139–150. doi:10.1007/978-1-62703-026-7_9.

Kozak, M. (1987). At least six nucleotides preceding the AUG initiator codon enhance translation in mammalian cells. *J. Mol. Biol.* 196, 947–950. doi:10.1016/0022-2836(87)90418-9.

Lasonder, E., Rijpma, S. R., Van Schaijk, B. C. L., Hoeijmakers, W. A. M., Kensche, P. R., Gresnigt, M. S., et al. (2016). Integrated transcriptomic and proteomic analyses of P. Falciparum gametocytes: Molecular insight into sex-specific processes and translational repression. *Nucleic Acids Res.* 44, 6087–6101. doi:10.1093/nar/gkw536.

Lemieux, J. E., Gomez-Escobar, N., Feller, A., Carret, C., Amambua-Ngwa, A., Pinches, R., et al. (2009). Statistical estimation of cell-cycle progression and lineage commitment in Plasmodium falciparum reveals a homogeneous pattern of transcription in ex vivo culture. *Proc. Natl. Acad. Sci. U. S. A.* 106, 7559–64. doi:10.1073/pnas.0811829106.

Malin, A. S., Content, J., Huygen, K., Andersen, P., and Dockrell, H. M. (1998). Vaccinia expression of Mycobacterium tuberculosis secreted proteins: The addition of tissue plasminogen activator signal sequence enhances expression and immunogenicity. *Thorax* 53.

Omondi, B. R., Muthui, M. K., Muasya, W. I., Orindi, B., Mwakubambanya, R. S., Bousema, T., et al. (2021). Antibody Responses to Crude Gametocyte Extract Predict Plasmodium falciparum Gametocyte Carriage in Kenya. *Front. Immunol.* 11, 1–14. doi:10.3389/fimmu.2020.609474.

Silvestrini, F., Bozdech, Z., Lanfrancotti, A., Di Giulio, E., Bultrini, E., Picci, L., et al. (2005). Genome-wide identification of genes upregulated at the onset of gametocytogenesis in Plasmodium falciparum. *Mol. Biochem. Parasitol.* 143, 100–110. doi:10.1016/j.molbiopara.2005.04.015.

Silvestrini, F., Lasonder, E., Olivieri, A., Camarda, G., van Schaijk, B., Sanchez, M., et al. (2010). Protein Export Marks the Early Phase of Gametocytogenesis of the Human Malaria Parasite Plasmodium falciparum. *Mol. Cell. Proteomics* 9, 1437–1448. doi:10.1074/mcp.M900479-MCP200.

Stone, W., , Joseph Campo , Isabelle Morlais, A. C., Drakely, C., Sutherland, C., , Douglas Molina, A. R., Pablo2, J., et al. (2015). Discovery of Novel Transmission Blocking Vaccine Candidates Using Gametocyte Protein Microarray. in *American Society of Tropical Medicine and Hygiene 64th Annual Meeting* (Philadelphia, Pennsylvania: American Journal of Tropical Medicine and Hygiene vol. 93 no. 4 Suppl 151-306), 167.

Tsuboi, T., Takeo, S., Sawasaki, T., Torii, M., and Endo, Y. (2010). “An Efficient Approach to the Production of Vaccines Against the Malaria Parasite,” in *Cell-Free Protein Production: Methods and Protocols*, eds. Y. Endo, K. Takai, and T. Ueda (Totowa, NJ: Humana Press), 73–83. doi:10.1007/978-1-60327-331-2_8.

Young, J. A., Fivelman, Q. L., Blair, P. L., De La Vega, P., Le Roch, K. G., Zhou, Y., et al. (2005). The Plasmodium falciparum sexual development transcriptome: A microarray analysis using ontology-based pattern identification. *Mol. Biochem. Parasitol.* 143, 67–79. doi:10.1016/j.molbiopara.2005.05.007.

# Supplementary Figures and Tables

## Supplementary Figures


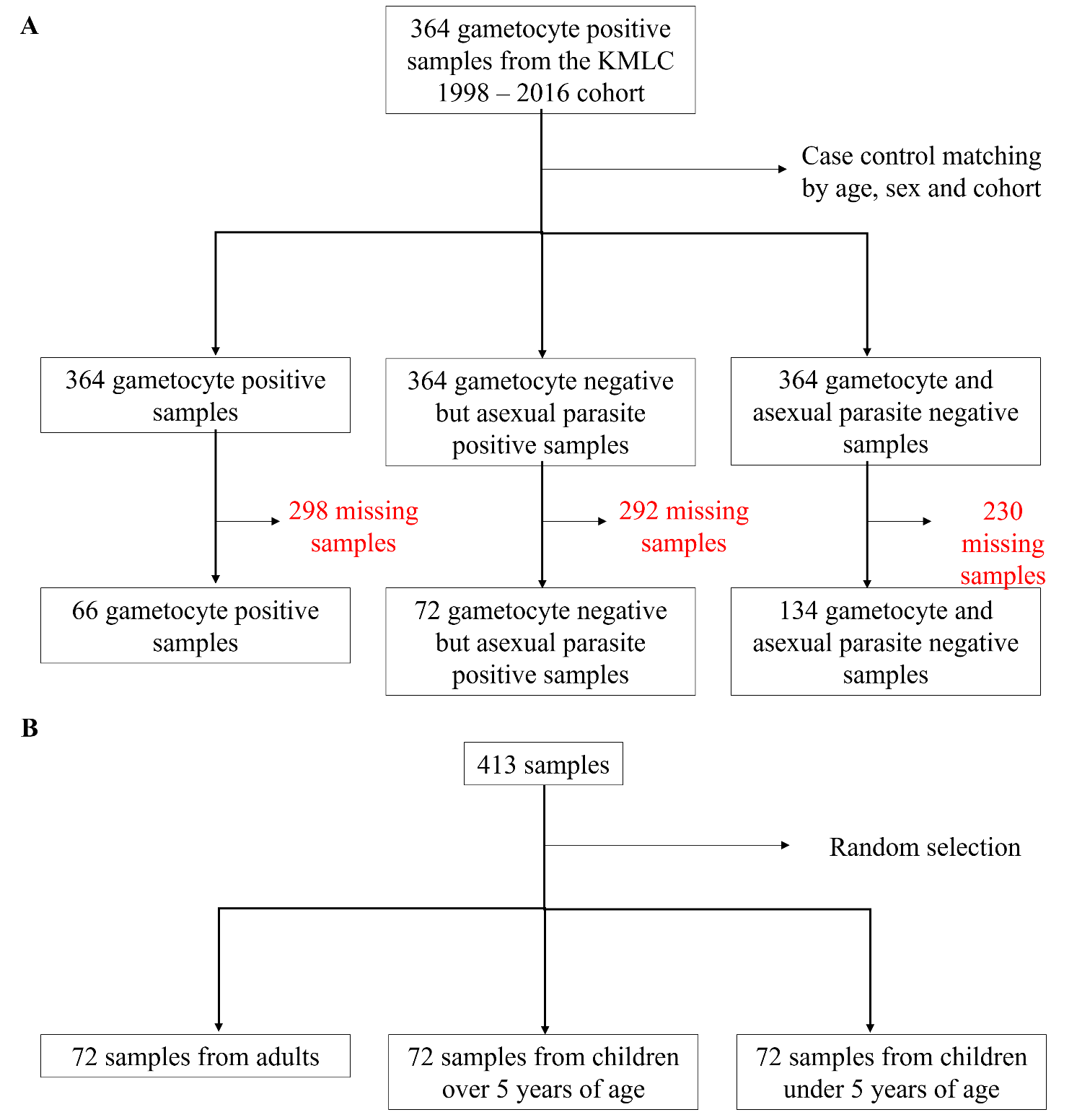


**Supplementary Figure 1.** Flow diagram showing the sample selection from immunoprofiling. A) Sample selection for the KMLC cohort for immunoprofiling of the mature gametocyte-stage proteins. B) Sample selection for the AFIRM cohort.


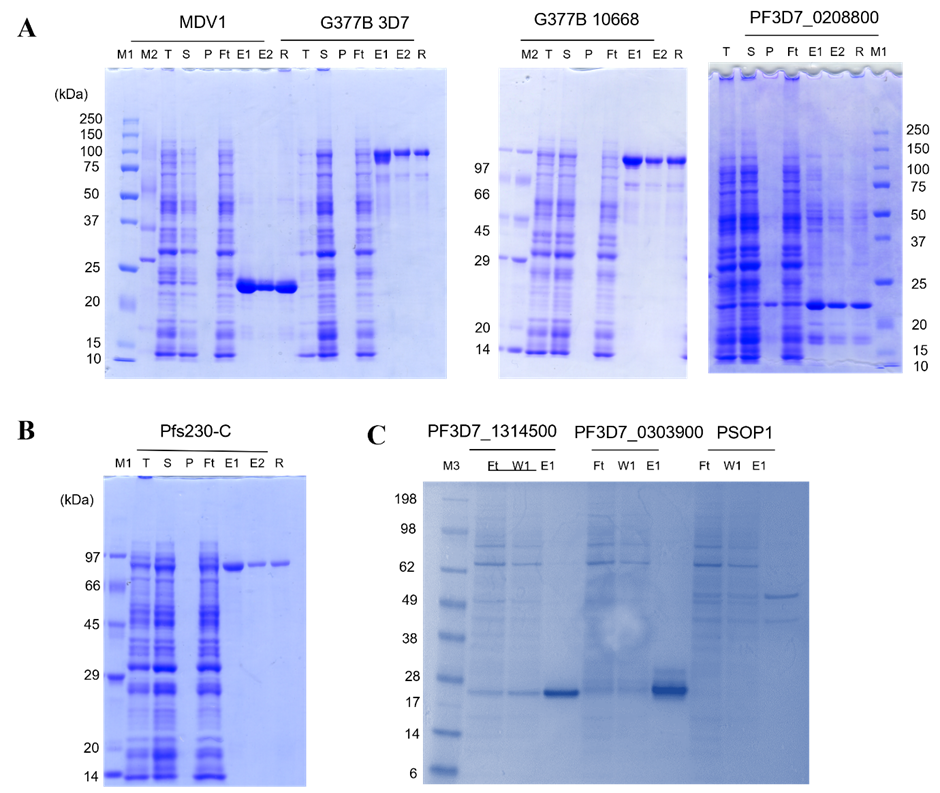


Supplementary Figure 2. Recombinant protein expression of the candidate antigens. The purified candidate antigens were generated by SDS-PAGE under reducing conditions, and the protein bands were visualized using Coomassie Blue staining. A) and B) Wheat germ cell-free system expressed proteins: MDV1 (22.9 kDa), G377B 3D7 (58.3 kDa), G377B – PfKE04 (59.3 kDa), PF3D7_0208800 (25.1 kDa), and Pfs230-C (79.6 kDa). The G377B variants migrate slower than expected on the SDS-PAGE gel. M1 – molecular weight marker 1 (Precision Plus Protein™ All Blue prestained protein standard (Bio-Rad)), M2 – molecular weight marker 2 (Low range protein standard (intégrale, Tokushima, Japan), T – total translation mix, S – supernatant. P – pellet, Ft - flow through, E – elution and R – resin. C) HEK293E expressed proteins: Pf3D7_1314500 (17.6 kDa), PF3D7_0303900 (20.4 kDa) and PSOP1 (51.4 kDa). M3 – molecular weight marker 3 – (SeeBlue™ Pre-stained Protein Standard (ThermoFisher Scientific)), Ft – flow though, W1 – first wash and E1 – first elution.


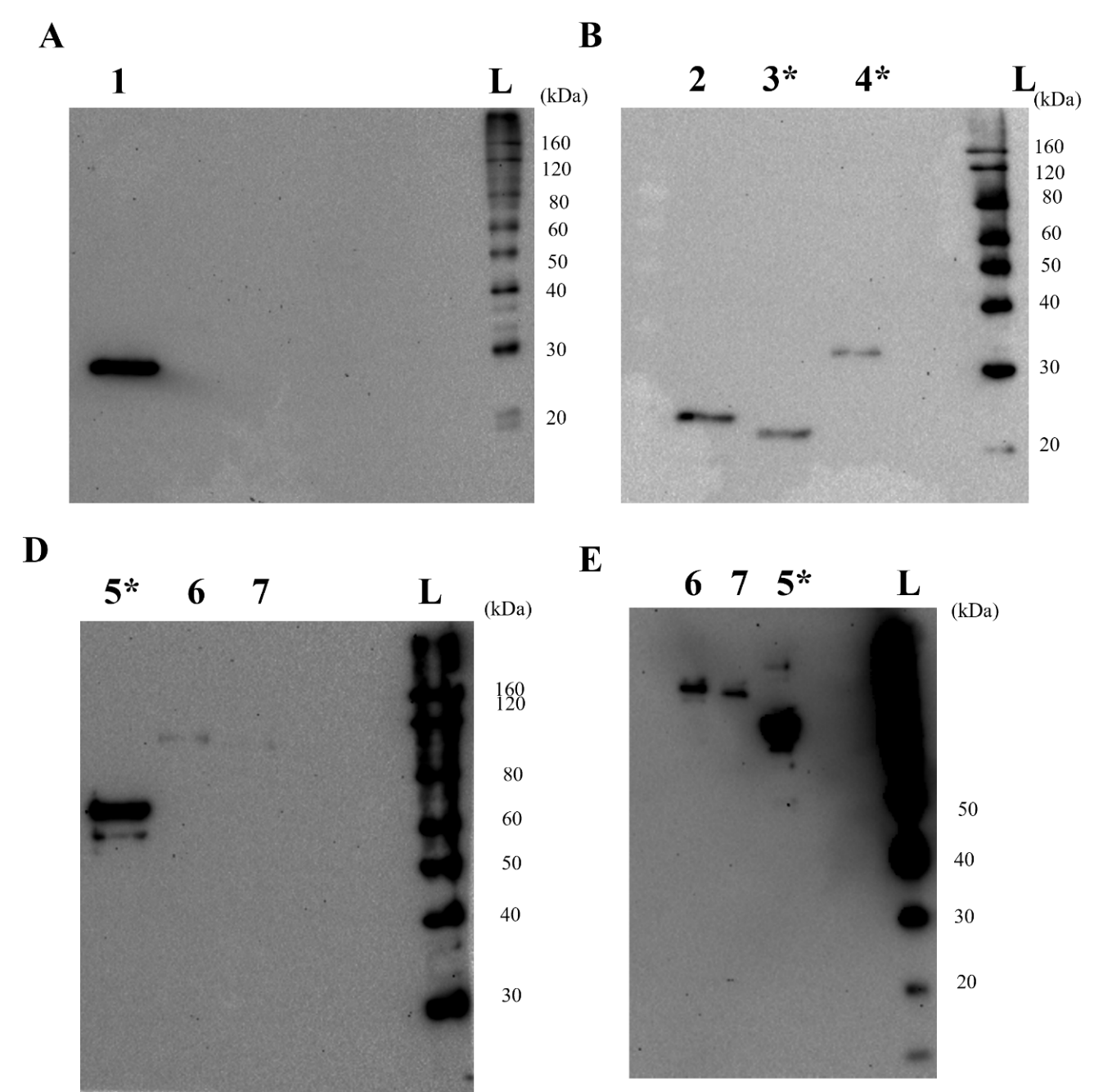


Supplementary Figure 3. Confirmatory western blot analysis of candidate antigens expressed in the wheat germ cell-free system (WGCFS). Proteins separated by SDS-PAGE were transferred onto PVDF membranes and probed with an antibody against the C-terminal hexahistidine tag. A) 1 – MDV1(22.9 kDa). B) 2 – PF3D7_0208800 (25.1 kDa), 3 – PF3D7_0303900 (20.4 kDa), 4 – PHISTa (32.2 kDa). * - PHISTa was not expressed in high enough quantities for use in the immunoprofiling analyses. Additionally, though PF3D7_0303900 was expressed in the WGCFS and HEK293E systems, large scale production was carried out in the HEK293E system. C) and D) 5 – PSOP25 PfKE04 (59.6 kDa), 6 – G377B 3D7 (58.3 kDa) and 7 – G377B PfKE04 (59.3 kDa). Western blot in panel E was taken after prolonged exposure to develop clearer protein bands for both G377B variants. The G377B variants migrate slower than expected on the SDS gel. *PSOP25 PfKE04 produced was not sufficient for use in the immunoprofiling analyses. La - BenchMark™ His-tagged Protein Standard (ThermoFisher Scientific).


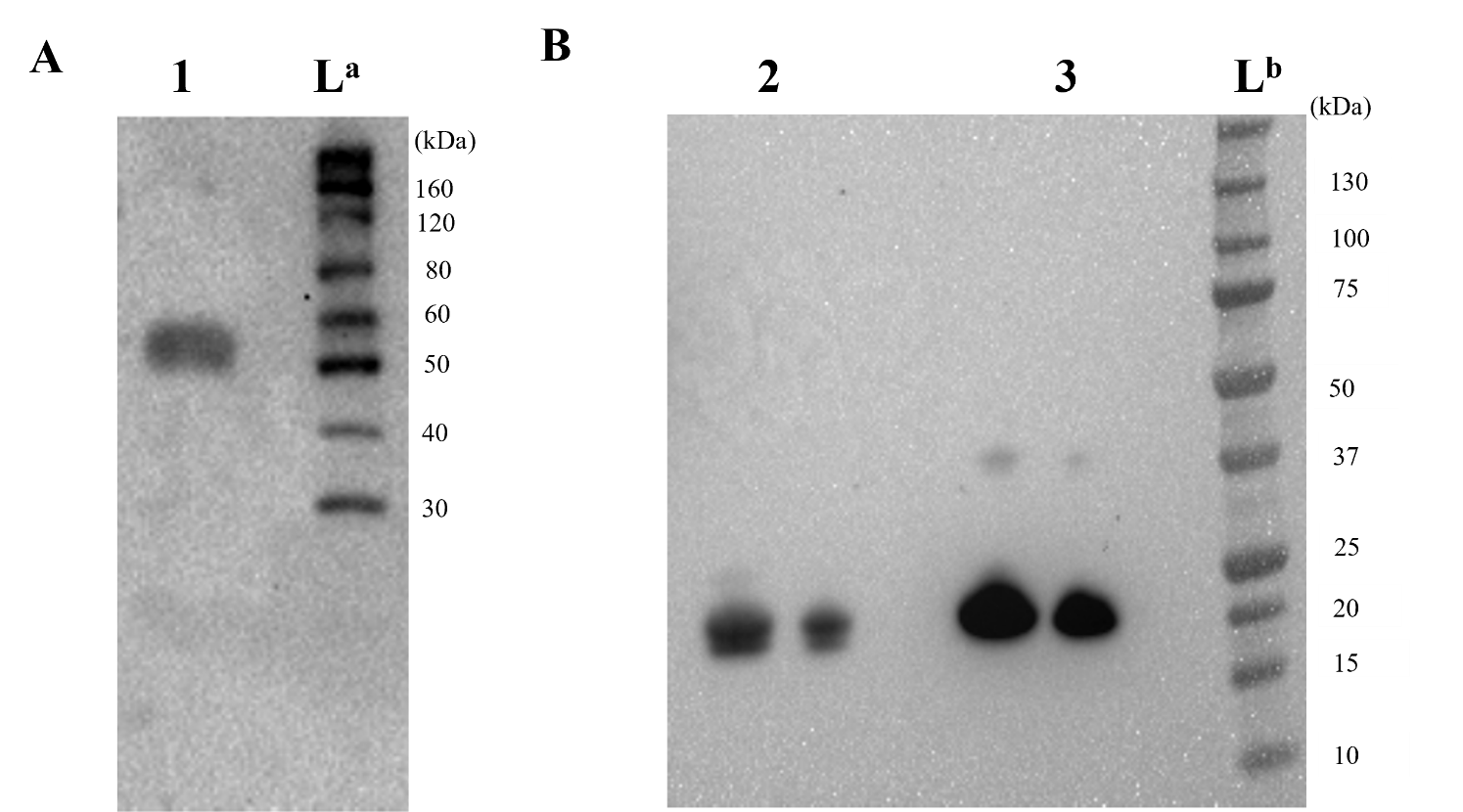


Supplementary Figure 4. Confirmatory western blot analysis of candidate antigens produced in the HEK293E system. Proteins separated by SDS-PAGE were transferred onto PVDF membranes and probed with an antibody against the C-terminal hexahistidine tag. A) 1 – PSOP1 (51.4 kDa). B) 2 – PF3D7_1314500 (17.6 kDa) and 3 – PF3D7_0303900 (20.4 kDa. La - BenchMark™ His-tagged Protein Standard (ThermoFisher Scientific) and Lb - SeeBlue™ Prestained Protein Standard (ThermoFisher Scientific).


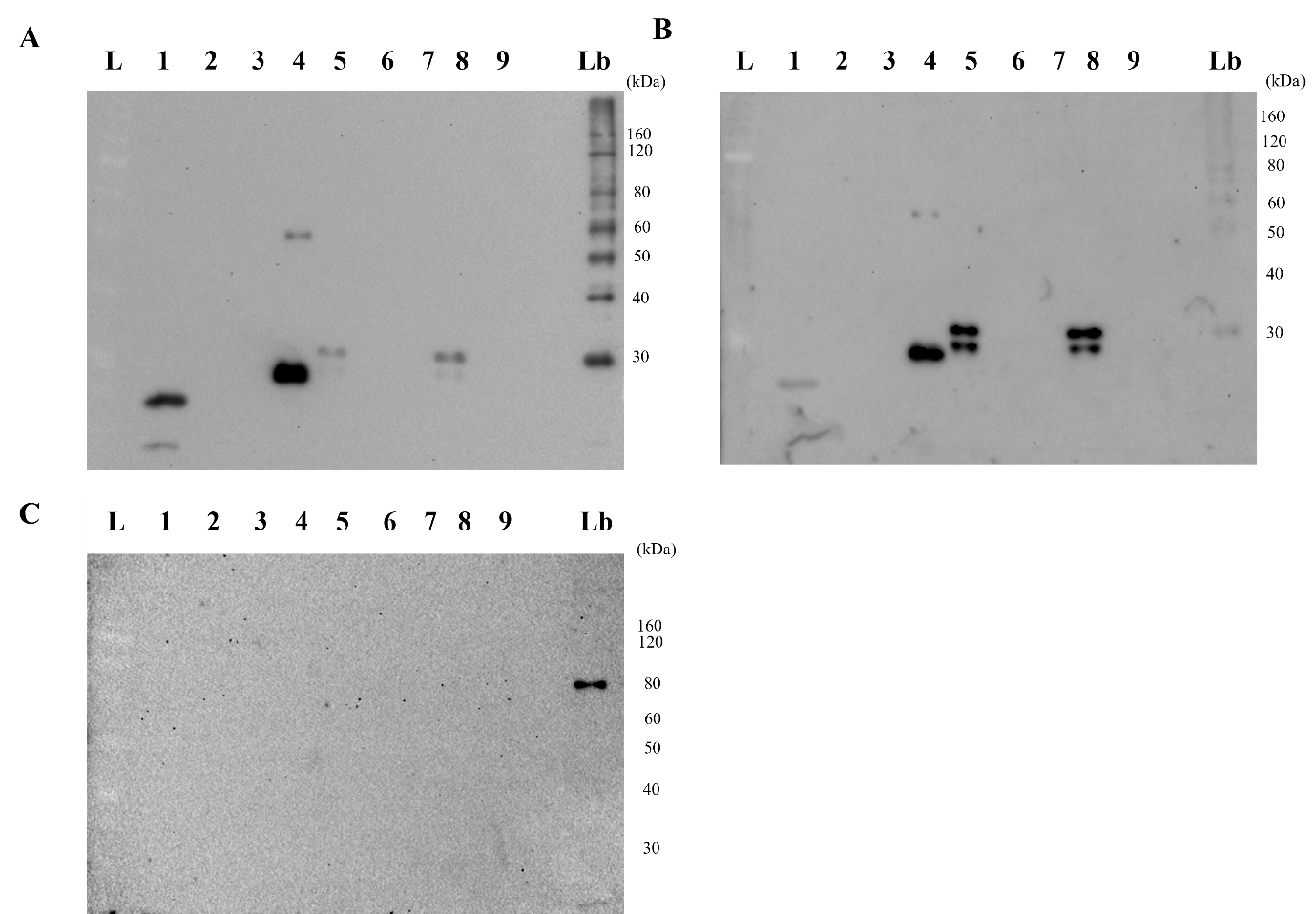


Supplementary Figure 5. Confirmatory western blot analysis of candidate antigens produced in the wheat germ cell-free system (WGCFS). Proteins separated by SDS-PAGE were transferred onto PVDF membranes and probed with an antibody against the C-terminal hexahistidine tag or with specific antisera. 1 – PF3D7_0309100* (21.4 kDa), 2 – PF3D7_1354400 (19.1 kDa), 3 – PF3D7_1346700 (46 kDa), 4 – MDV1 (25.8 kDa), 5 – PF3D7_1105800 PfKE04* (30.7 kDa), 6 - PF3D7_1314500 (17.6 kDa), 7 - PSOP1 (51.4 kDa), 8 – PF3D7_1105800 3D7* (30.7 kDa), 9 - MOCK – wheat germ extract purified in the same way as the expressed proteins included as a negative control. A) Western blot showing recognition of PF3D7_0309100, MDV1 and PF3D7_1105800 when probed with the anti-histidine tag antibody. B) Western blot showing recognition of PF3D7_0309100, MDV1 and PF3D7_1105800 by a random pool of sera from malaria-exposed individuals (AFIRM cohort). C) Western blot showing no recognition of the gametocyte antigens when probed with sera from malaria naïve individuals. La - Color Prestained Protein Standard, Broad Range (New England BioLabs) and Lb - BenchMark™ His-tagged Protein Standard (ThermoFisher Scientific). *PF3D7_0309100 and PF3D7_1105800 were not produced at high enough yield for use in the immunoprofiling analyses.


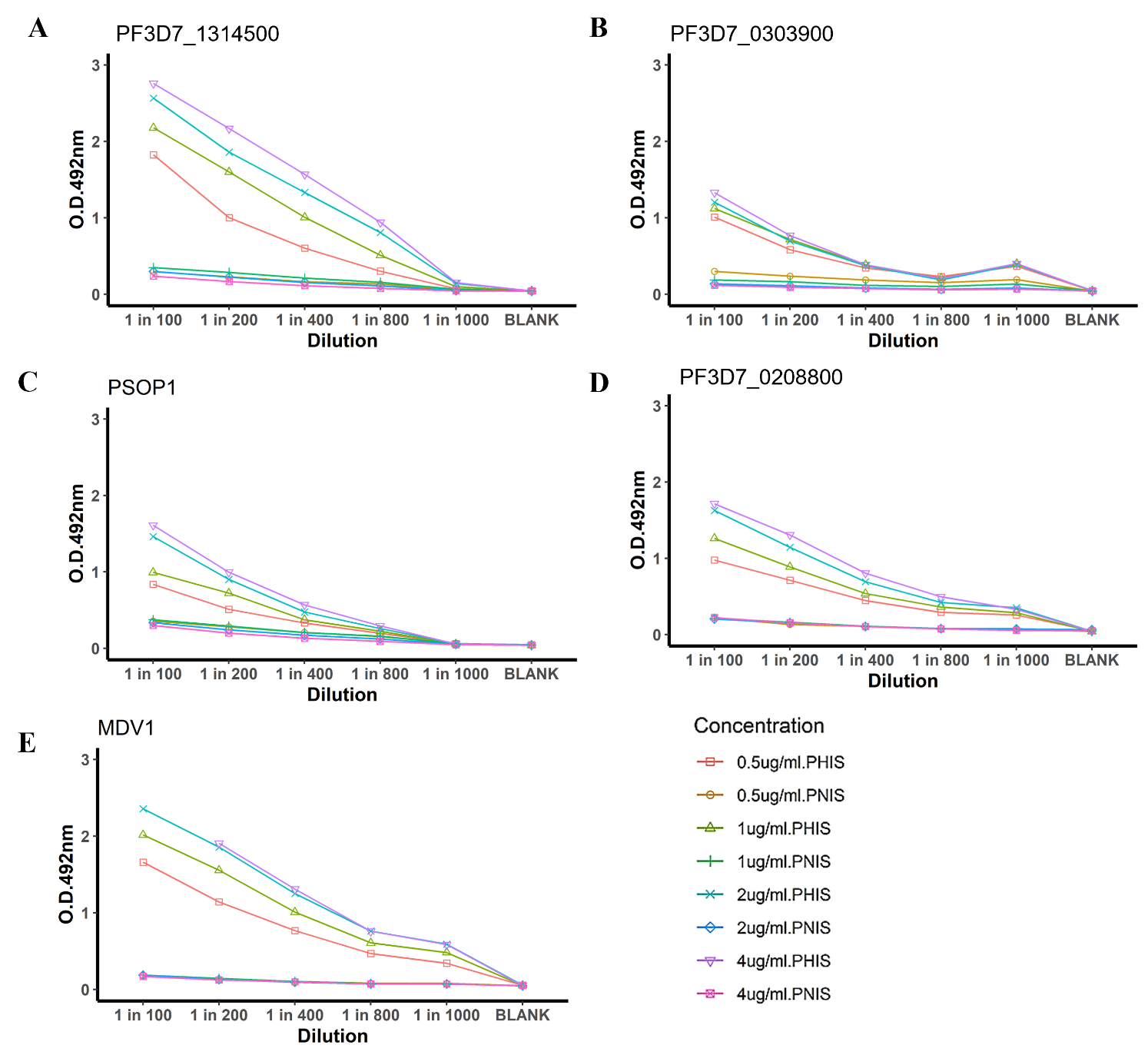


**Supplementary Figure 6. Checkerboard titrations to determine optimal antigen concentration and serum dilutions for ELISA.** Line plots showing the titration of different antigen concentrations of the gametocyte antigens against dilutions of pooled hyperimmune sera (PHIS) or pooled non-immune sera (PNIS). O.D.492nm – optical density read at 492 nm.


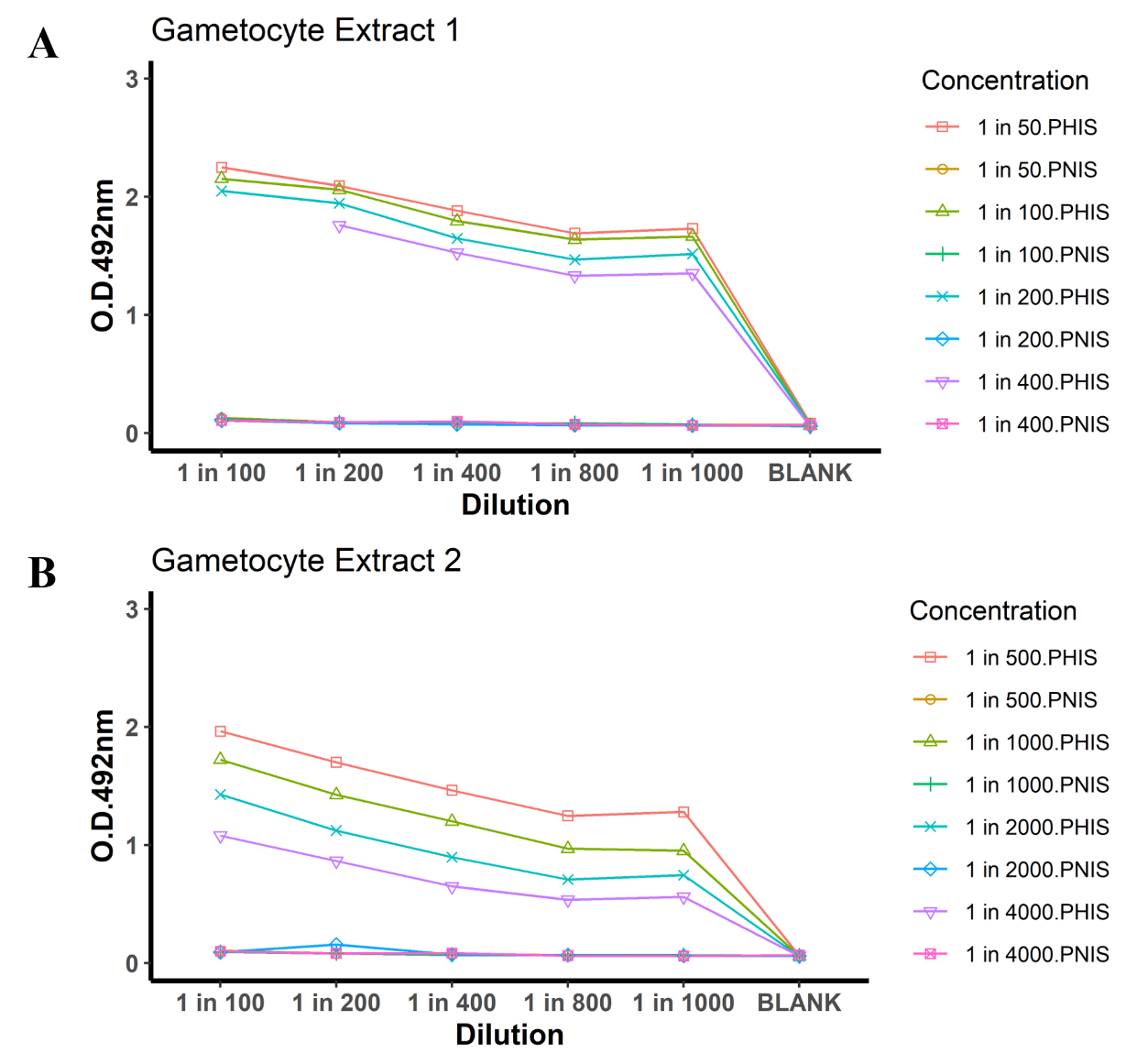


**Supplementary Figure 7. Checkerboard titrations to determine optimal gametocyte extract dilution and serum dilution for ELISA.** Line plots showing the titration of different antigen concentrations of the gametocyte antigens against dilutions of pooled hyperimmune sera (PHIS) or pooled non-immune sera (PNIS). O.D.492nm – optical density read at 492 nm.


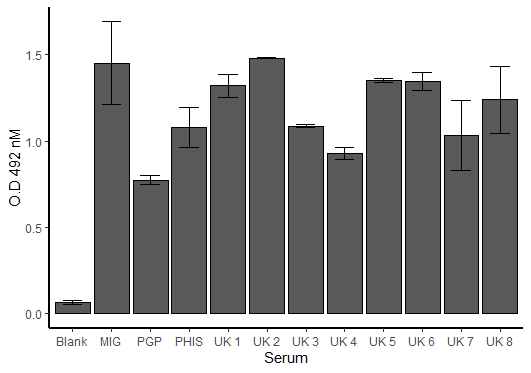


Supplementary Figure 8. Test of the negative control serum (non-immune serum, NIS) against the measles antigen. Barplots showing reactivity of the NIS (UK 1 – 8) and a panel of positive controls to 0.9 μg/ml of measles recombinant antigen. These NIS are malaria naïve but would be expected to react to the measles antigen as we expect the donors to have received the measles vaccine in childhood. Positive controls - PHIS – pooled hyperimmune serum, PGP – pooled gametocyte positive sera, MIG – malaria immune globulin. Blank represents wells with only secondary antibody added (no antigen or test sera) to correct for background.


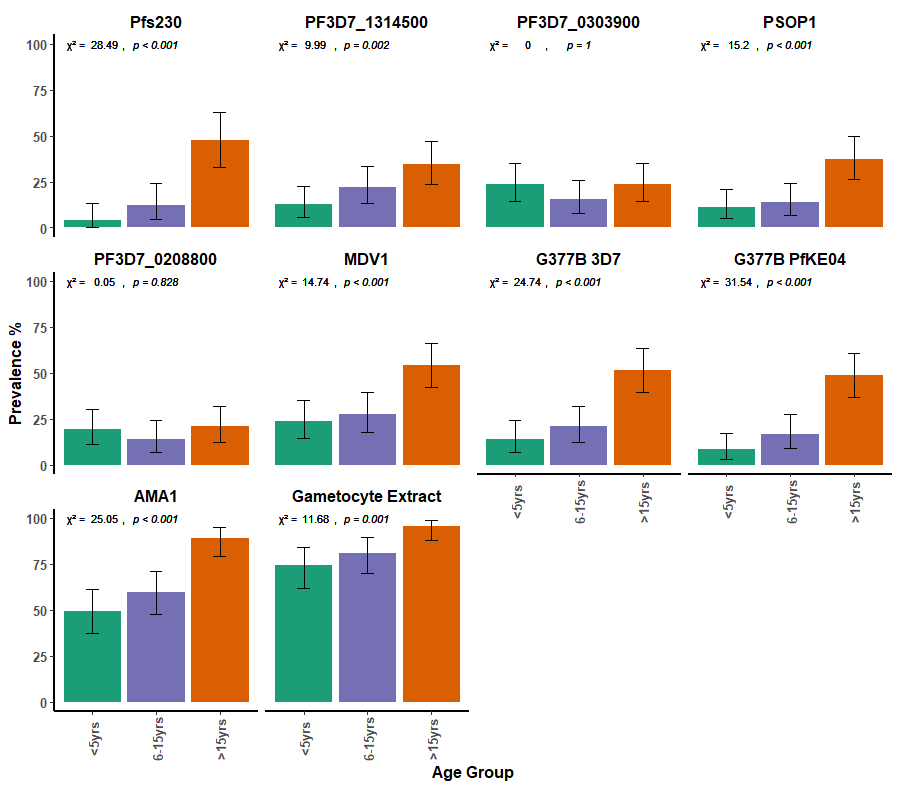


**Supplementary Figure 9.** **Seroprevalence to the candidate antigens, AMA1 and gametocyte extract stratified by age group in the AFIRM Cohort.** Bar plots showing the prevalence of antibodies to the candidate antigens, AMA1 and gametocyte extract within the different age categories. Seroprevalence was calculated relative to a statistically determined subset of low responders. The Cochran-Armitage test for trend was used to analyze the relationship between seroprevalence and age; *p* values are presented at the top of each panel. Error bars show 95% binomial confidence intervals (Clopper–Pearson interval).


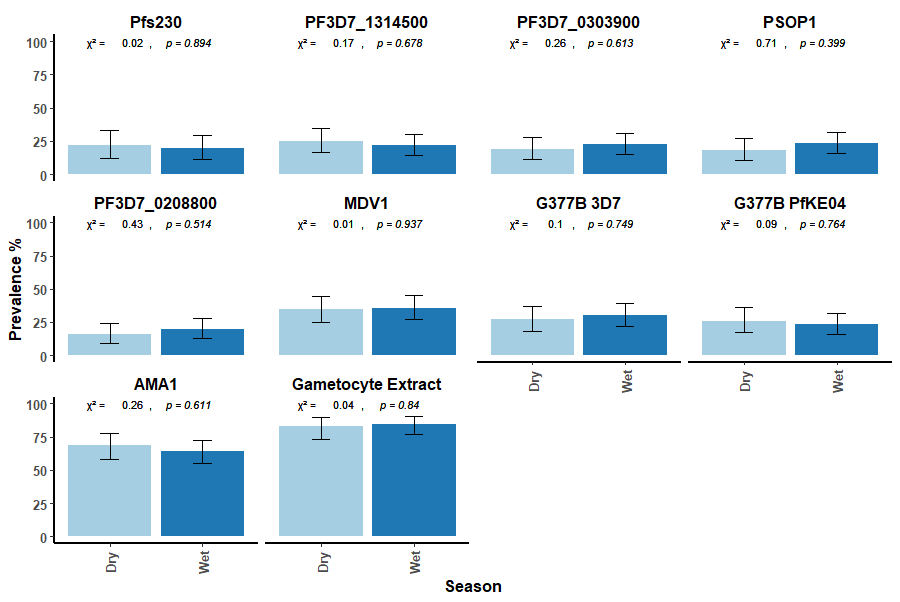


**Supplementary Figure 10. Seroprevalence to the candidate antigens, AMA1 and gametocyte extract stratified by season in the AFIRM cohort.** Bar plots showing the prevalence of antibodies to the candidate antigens, AMA1 and gametocyte extract in the dry and the wet seasons. Seroprevalence was calculated relative to a statistically determined subset of low responders. Chi-square analysis was used to compare proportions in the dry and wet seasons. Respective *p* values are presented at the top of each panel. Error bars show 95% binomial confidence intervals (Clopper–Pearson interval).


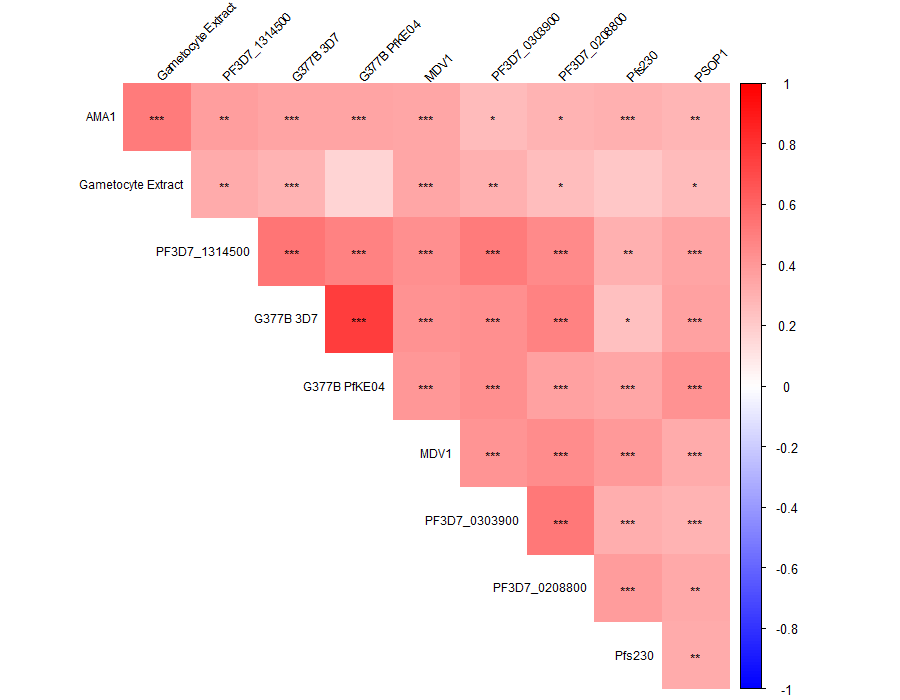


**Supplementary Figure 11. Correlation matrix of seropositivity to the candidate antigens, AMA1 and gametocyte extract.** The dependency between seropositivity to the different antigens was tested by Spearman’s rank correlation. The asterisks within the boxes of the matrices indicate the significance of the association, with *p* < 0.0001 as ***, *p* <0.001 as ** and *p* < 0.01 as *.


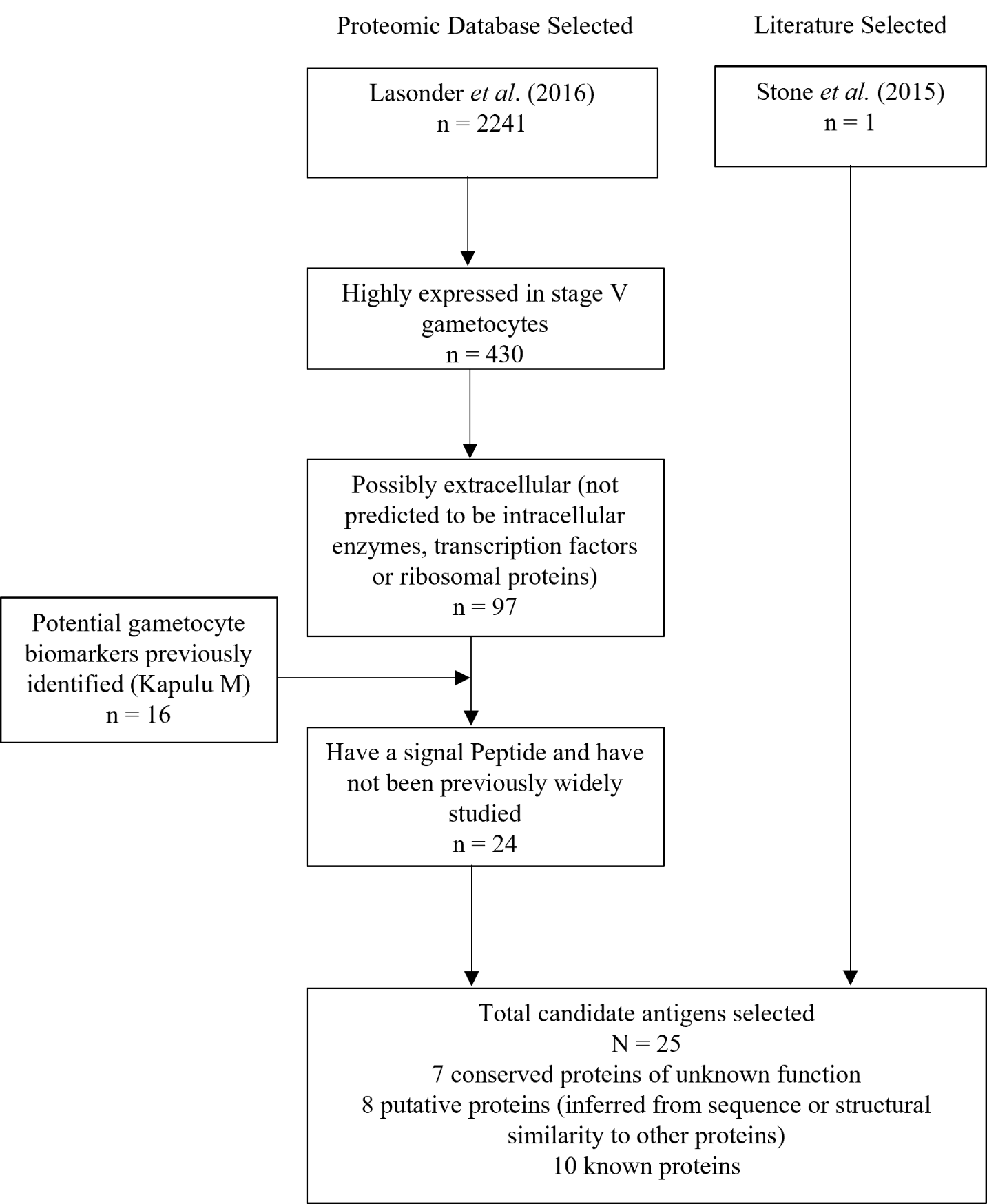


Supplementary Figure 12: Flow diagram showing the selection of candidate gametocyte antigens for study. The number of proteins at each step is also indicated.

## Supplementary Tables

**Supplementary Table 2: 2 x 2 tables of seropositivity to the G377 variants**

|  |  | **G377B PfKE04** | | |  |
| --- | --- | --- | --- | --- | --- |
|  |  | **Seronegative** | **Seropositive** | **Total** |  |
| **G377B 3D7** | **Seronegative** | **44** | **4** | **48** |  |
|  | **Seropositive** | **20** | **148** | **168** |  |
|  | **Total** | **64** | **152** | **216** |  |

**Supplementary Table 3: Seroprevalence of immune responses to the gametocyte antigens, AMA1 and gametocyte extract as estimated relative to a statistically defined subset of low responders**

| **Gene ID** | **Gene name^a^** | **N^b^** | **Seropositive** | **Prevalence (95% CI)** |
| --- | --- | --- | --- | --- |
| PF3D7_0209000 | P230 | 148 | 30 | 20.3 (14.1, 27.7) |
| PF3D7_1314500 | - | 216 | 50 | 23.1 (17.7, 29.4) |
| PF3D7_0303900 | - | 216 | 45 | 20.8 (15.6, 26.9) |
| PF3D7_0721700 | PSOP1 | 216 | 45 | 20.8 (15.6, 26.9) |
| PF3D7_0208800 | - | 216 | 39 | 18.1 (13.2, 23.8) |
| PF3D7_1216500 | MDV1 | 216 | 76 | 35.2 (28.8, 42.0) |
| PF3D7_1250100 | G377B 3D7 | 216 | 62 | 28.7 (22.8, 35.2) |
| - | G377B PfKE04 | 216 | 53 | 24.5 (19.0, 30.8) |
| PF3D7_1133400 | AMA1 | 215 | 142 | 66.0 (59.2, 72.3) |
| - | Gametocyte Extract | 205 | 172 | 83.9 (78.1, 88.7) |

**Supplementary Table 4: Spearman’s rank correlation coefficients from a correlation matrix of seropositivity to the candidate antigens, AMA1 and gametocyte extract**

|  | **Pfs230** | **PF3D7_13145000** | **PF3D7_0303900** | **PSOP1** | **G377B 3D7** | **G377B PfKE04** | **PF3D7_0208800** | **MDV1** | **AMA1** | **Gametocyte Extract** |
| --- | --- | --- | --- | --- | --- | --- | --- | --- | --- | --- |
| **Pfs230** | 1 | 0.3 | 0.32 | 0.33 | 0.25 | 0.34 | 0.38 | 0.39 | 0.3 | 0.21 |
| **PF3D7_13145000** |  | 1 | 0.52 | 0.36 | 0.53 | 0.48 | 0.45 | 0.43 | 0.37 | 0.33 |
| **PF3D7_0303900** |  |  | 1 | 0.3 | 0.44 | 0.44 | 0.52 | 0.41 | 0.26 | 0.31 |
| **PSOP1** |  |  |  | 1 | 0.36 | 0.43 | 0.34 | 0.33 | 0.28 | 0.26 |
| **G377B 3D7** |  |  |  |  | 1 | 0.76 | 0.49 | 0.43 | 0.36 | 0.3 |
| **G377B PfKE04** |  |  |  |  |  | 1 | 0.36 | 0.41 | 0.35 | 0.16 |
| **PF3D7_0208800** |  |  |  |  |  |  | 1 | 0.44 | 0.3 | 0.25 |
| **MDV1** |  |  |  |  |  |  |  | 1 | 0.35 | 0.35 |
| **AMA1** |  |  |  |  |  |  |  |  | 1 | 0.51 |
| **Gametocyte Extract** |  |  |  |  |  |  |  |  |  | 1 |

Supplementary Table 5: Univariable linear regression analysis of the factors predicting the magnitude of antibody response to the gametocyte antigens, AMA1 and gametocyte extract – KMLC cohort

| **Covariate** | **Pfs230** | | **PF3D7_1314500** | | **PF3D7_0303900** | | **PSOP1** | |
| --- | --- | --- | --- | --- | --- | --- | --- | --- |
|  | **Estimate**  **(95% CI)** | ***p* value** | **Estimate**  **(95% CI)** | ***p* value** | **Estimate**  **(95% CI)** | ***p* value** | **Estimate**  **(95% CI)** | ***p* value** |
| Age Group | Ref. | . | Ref. | . | Ref. | . | Ref. | . |
| 0 - 5 years | 0.15 (0.05, 0.26) | **0.0032** | 0.08 (-0.03, 0.19) | 0.1728 | 0.05 (-0.07, 0.16) | 0.4296 | 0.06 (-0.05, 0.18) | 0.2728 |
| 6 - 10 years | 0.21 (0.05, 0.36) | **0.0075** | 0.11 (-0.08, 0.3) | 0.2546 | 0.01 (-0.16, 0.17) | 0.9374 | 0.14 (-0.07, 0.35) | 0.1974 |
| 11 - 15 years | 0.24 (0.15, 0.34) | **<0.001** | 0.31 (0.21, 0.42) | **<0.001** | 0.34 (0.24, 0.44) | **<0.001** | 0.21 (0.10, 0.32) | **<0.001** |
| Asexual parasite positive | 0.09 (-0.01, 0.19) | 0.0855 | 0.29 (0.17, 0.42) | **<0.001** | 0.28 (0.16, 0.41) | **<0.001** | 0.15 (0.02, 0.28) | **0.0254** |
| Gametocyte positive |  |  |  |  |  |  |  |  |
| Sickle | Ref. | . | Ref. | . | Ref. | . | Ref. | . |
| Normal | 0.09 (-0.04, 0.21) | 0.1590 | 0.02 (-0.11, 0.15) | 0.7467 | 0.08 (-0.04, 0.2) | 0.1847 | 0.08 (-0.06, 0.23) | 0.2583 |
| Heterozygous |  |  |  |  |  |  |  |  |
| α - Thalassaemia |  |  |  |  |  |  |  |  |
| Normal | Ref. | . | Ref. | . | Ref. | . | Ref. | . |
| Heterozygous | -0.03 (-0.14, 0.07) | 0.5248 | 0.02 (-0.11, 0.14) | 0.7609 | -0.09 (-0.21, 0.03) | 0.1435 | -0.03 (-0.16, 0.1) | 0.6278 |
| Homozygous | -0.04 (-0.2, 0.11) | 0.5603 | -0.11 (-0.25, 0.04) | 0.1568 | -0.06 (-0.21, 0.09) | 0.4526 | 0.10 (-0.10, 0.29) | 0.3332 |
| Cohort |  |  |  |  |  |  |  |  |
| Junju | Ref. | . | Ref. | . | Ref. | . | Ref. | . |
| Ngerenya-early | -0.13 (-0.27, 0.02) | 0.0868 | 0.04 (-0.13, 0.21) | 0.6162 | 0.05 (-0.11, 0.22) | 0.5226 | 0.03 (-0.14, 0.21) | 0.7055 |
| Ngerenya-late | -0.16 (-0.26, -0.05) | **0.0032** | -0.17 (-0.29, -0.05) | **0.0041** | -0.17 (-0.28, -0.05) | **0.0048** | -0.14 (-0.27, -0.01) | **0.0297** |
| **Covariate** | **PF3D7_0208800** | | **MDV1** | | **G377B 3D7** | | **G3D7 PfKE04** | |
|  | **Estimate**  **(95% CI)** | ***p* value** | **Estimate**  **(95% CI)** | ***p* value** | **Estimate**  **(95% CI)** | ***p* value** | **Estimate**  **(95% CI)** | ***p* value** |
| Age Group |  |  |  |  |  |  |  |  |
| 0 - 5 years | Ref. | . | Ref. | . | Ref. | . | Ref. | . |
| 6 - 10 years | 0.07 (-0.03, 0.17) | 0.1470 | 0.09 (-0.02, 0.19) | 0.1051 | 0.20 (0.09, 0.31) | **<0.001** | 0.22 (0.11, 0.32) | **<0.001** |
| 11 - 15 years | 0.0004 (-0.14, 0.14) | 0.9950 | 0.14 (-0.10, 0.38) | 0.2431 | 0.25 (0.06, 0.44) | **0.0092** | 0.28 (0.10, 0.46) | **0.0021** |
|  |  | |  | |  | |  | |
| **Covariate** | **PF3D7_0208800** | | **MDV1** | | **G377B 3D7** | | **G3D7 PfKE04** | |
|  | **Estimate**  **(95% CI)** | ***p* value** | **Estimate**  **(95% CI)** | ***p* value** | **Estimate**  **(95% CI)** | ***p* value** | **Estimate**  **(95% CI)** | ***p* value** |
| Asexual parasite positive | 0.21 (0.12, 0.29) | **<0.001** | 0.24 (0.14, 0.35) | **<0.001** | 0.33 (0.22, 0.43) | **<0.001** | 0.30 (0.20, 0.40) | **<0.001** |
| Gametocyte positive | 0.20 (0.09, 0.30) | **<0.001** | 0.25 (0.13, 0.36) | **<0.001** | 0.22 (0.11, 0.34) | **<0.001** | 0.20 (0.09, 0.30) | **<0.001** |
| Sickle |  |  |  |  |  |  |  |  |
| Normal | Ref. | . | Ref. | . | Ref. | . | Ref. | . |
| Heterozygous | 0.02 (-0.09, 0.14) | 0.6794 | -0.08 (-0.20, 0.05) | 0.2145 | 0.08 (-0.07, 0.23) | 0.2859 | 0.09 (-0.06, 0.25) | 0.2347 |
| α - Thalassaemia |  |  |  |  |  |  |  |  |
| Normal | Ref. | . | Ref. | . | Ref. | . | Ref. | . |
| Heterozygous | -0.03 (-0.13, 0.06) | 0.5222 | -0.02 (-0.14, 0.1) | 0.7933 | 0.01 (-0.11, 0.13) | 0.8603 | -0.01 (-0.12, 0.10) | 0.8417 |
| Homozygous | -0.07 (-0.21, 0.07) | 0.3301 | -0.15 (-0.29, -0.01) | **0.0408** | -0.06 (-0.23, 0.12) | 0.5021 | -0.10 (-0.28, 0.09) | 0.2992 |
| Cohort |  |  |  |  |  |  |  |  |
| Junju | Ref. | . | Ref. | . | Ref. | . | Ref. | . |
| Ngerenya-early | -0.06 (-0.19, 0.08) | 0.4138 | 0.06 (-0.12, 0.24) | 0.5205 | -0.09 (-0.25, 0.07) | 0.2676 | -0.10 (-0.25, 0.06) | 0.2179 |
| Ngerenya-late | -0.11 (-0.21, -0.01) | **0.0311** | -0.14 (-0.25, -0.03) | **0.0154** | -0.27 (-0.39, -0.15) | **<0.001** | -0.25 (-0.38, -0.13) | **<0.001** |
| **Covariate** | **AMA1** | | **GE** | |  |  |  |  |
|  | **Estimate**  **(95% CI)** | ***p* value** | **Estimate**  **(95% CI)** | ***p* value** |  |  |  |  |
| Age Group |  |  |  |  |  |  |  |  |
| 0 - 5 years | Ref. | . | Ref. | . |  |  |  |  |
| 6 - 10 years | 0.39 (0.08, 0.69) | **0.0129** | 0.12 (-0.1, 0.33) | 0.2819 |  |  |  |  |
| 11 - 15 years | 0.69 (0.2, 1.19) | **0.0060** | 0.35 (0, 0.7) | **0.0471** |  |  |  |  |
| Asexual parasite positive | 1.24 (0.99, 1.48) | **<0.001** | 0.68 (0.5, 0.87) | **<0.001** |  |  |  |  |
| Gametocyte positive | 0.92 (0.64, 1.2) | **<0.001** | 0.55 (0.36, 0.74) | **<0.001** |  |  |  |  |
| Sickle |  |  |  |  |  |  |  |  |
| Normal | Ref. | . | Ref. | . |  |  |  |  |
| Heterozygous | 0.08 (-0.26, 0.43) | 0.6315 | 0.23 (0, 0.45) | **0.0489** |  |  |  |  |
| α - Thalassaemia |  |  |  |  |  |  |  |  |
| Normal | Ref. | . | Ref. | . |  |  |  |  |
| Heterozygous | -0.25 (-0.57, 0.08) | 0.3198 | -0.32 (-0.55, -0.09) | **0.0065** |  |  |  |  |
| Homozygous | -0.23 (-0.68, 0.22) | 0.1413 | -0.36 (-0.65, -0.06) | **0.0161** |  |  |  |  |
| **Covariate** | **Pfs230** | | **PF3D7_1314500** | |  |  |  |  |
|  | **Estimate**  **(95% CI)** | ***p* value** | **Estimate**  **(95% CI)** | ***p* value** |  |  |  |  |
| Cohort |  |  |  |  |  |  |  |  |
| Junju | Ref. | . | Ref. | . |  |  |  |  |
| Ngerenya-early | 0.20 (-0.19, 0.58) | 0.3168 | -0.09 (-0.37, 0.20) | 0.5537 |  |  |  |  |
| Ngerenya-late | -0.83 (-1.14, -0.51) | **<0.001** | -0.62 (-0.83, -0.41) | **<0.001** |  |  |  |  |

Ref. – reference category. *P* values in bold are statistically significant (*p* <0.05).

Supplementary Table 6: Multivariable linear regression analysis of the factors predicting the magnitude of antibody response to the gametocyte antigens, AMA1 and gametocyte extract – KMLC cohort

| **Covariate** | **Pfs230** | | **PF3D7_1314500** | | **PF3D7_0303900** | | **PSOP1** | |
| --- | --- | --- | --- | --- | --- | --- | --- | --- |
|  | **Estimate**  **(95% CI)** | ***p* value** | **Estimate**  **(95% CI)** | ***p* value** | **Estimate**  **(95% CI)** | ***p* value** | **Estimate**  **(95% CI)** | ***p* value** |
| Age Group |  |  |  |  |  |  |  |  |
| 0 - 5 years | Ref. | . | Ref. | . | Ref. | . | Ref. | . |
| 6 - 10 years | 0.15 (0.04, 0.25) | **0.0056** | 0.06 (-0.05, 0.17) | 0.2857 | 0.02 (-0.09, 0.14) | 0.6728 | 0.05 (-0.07, 0.17) | 0.4236 |
| 11 - 15 years | 0.17 (0.03, 0.31) | **0.0193** | 0.06 (-0.13, 0.25) | 0.5172 | -0.02 (-0.18, 0.14) | 0.8106 | 0.13 (-0.08, 0.34) | 0.2312 |
| Asexual parasite positive | 0.25 (0.12, 0.39) | **<0.001** | 0.28 (0.14, 0.42) | **<0.001** | 0.28 (0.14, 0.42) | **<0.001** | 0.17 (0.01, 0.33) | **0.0418** |
| Gametocyte positive | 0.02 (-0.11, 0.15) | 0.7498 | 0.18 (0.03, 0.34) | **0.0225** | 0.17 (0.01, 0.33) | **0.0353** | 0.08 (-0.09, 0.24) | 0.352 |
| Sickle |  |  |  |  |  |  |  |  |
| Normal | Ref. | . | Ref. | . | Ref. | . | Ref. | . |
| Heterozygous | -0.002 (-0.12, 0.12) | 0.9801 | -0.01 (-0.14, 0.11) | 0.8159 | 0.05 (-0.07, 0.18) | 0.4088 | 0.06 (-0.08, 0.21) | 0.3977 |
| α - Thalassaemia |  |  |  |  |  |  |  |  |
| Normal | Ref. | . | Ref. | . | Ref. | . | Ref. | . |
| Heterozygous | -0.01 (-0.12, 0.09) | 0.8245 | 0.06 (-0.06, 0.18) | 0.2926 | -0.04 (-0.15, 0.07) | 0.5284 | -0.01 (-0.13, 0.11) | 0.9114 |
| Homozygous | -0.03 (-0.17, 0.11) | 0.6879 | -0.05 (-0.20, 0.09) | 0.4653 | -0.01 (-0.16, 0.14) | 0.9069 | 0.13 (-0.07, 0.33) | 0.2082 |
| Cohort |  |  |  |  |  |  |  |  |
| Junju | Ref. | . | Ref. | . | Ref. | . | Ref. | . |
| Ngerenya-early | -0.15 (-0.30, 0.01) | 0.0679 | -0.05 (-0.24, 0.14) | 0.5965 | -0.03 (-0.21, 0.14) | 0.7291 | 0.04 (-0.16, 0.24) | 0.6904 |
| Ngerenya-late | -0.03 (-0.15, 0.09) | 0.6353 | -0.001 (-0.13, 0.13) | 0.9848 | 0.004 (-0.12, 0.13) | 0.9530 | -0.03 (-0.19, 0.13) | 0.7003 |
| **Covariate** | **PF3D7_0208800** | | **MDV1** | | **G377B 3D7** | | **G3D7 PfKE04** | |
|  | **Estimate**  **(95% CI)** | ***p* value** | **Estimate**  **(95% CI)** | ***p* value** | **Estimate**  **(95% CI)** | ***p* value** | **Estimate**  **(95% CI)** | ***p* value** |
| Age Group |  |  |  |  |  |  |  |  |
| 0 - 5 years | Ref. | . | Ref. | . | Ref. | . | Ref. | . |
| 6 - 10 years | 0.08 (-0.02, 0.17) | 0.1297 | 0.10 (-0.004, 0.21) | 0.0579 | 0.20 (0.09, 0.31) | **<0.001** | 0.21 (0.11, 0.32) | **<0.001** |
| 11 - 15 years | -0.01 (-0.14, 0.12) | 0.8783 | 0.12 (-0.12, 0.37) | 0.3194 | 0.19 (0.03, 0.35) | **0.0173** | 0.24 (0.08, 0.39) | **0.0033** |
|  |  |  |  |  |  |  |  |  |
|  |  |  |  |  |  |  |  |  |
| **Covariate** | **PF3D7_0208800** | | **MDV1** | | **G377B 3D7** | | **G3D7 PfKE04** | |
|  | **Estimate**  **(95% CI)** | ***p* value** | **Estimate**  **(95% CI)** | ***p* value** | **Estimate**  **(95% CI)** | ***p* value** | **Estimate**  **(95% CI)** | ***p* value** |
| Asexual parasite positive | 0.19 (0.07, 0.31) | **0.0014** | 0.20 (0.07, 0.34) | **0.0033** | 0.27 (0.15, 0.39) | **<0.001** | 0.25 (0.13, 0.37) | **<0.001** |
| Gametocyte positive | 0.15 (0.01, 0.28) | **0.0318** | 0.16 (-0.01, 0.32) | **0.0592** | 0.1 (-0.02, 0.23) | 0.1091 | 0.09 (-0.03, 0.22) | 0.1516 |
| Sickle |  |  |  |  |  |  |  |  |
| Normal | Ref. | . | Ref. | . | Ref. | . | Ref. | . |
| Heterozygous | -0.02 (-0.14, 0.09) | 0.7038 | -0.11 (-0.23, 0.01) | 0.0689 | -0.001 (-0.14, 0.13) | 0.9554 | 0.01 (-0.13, 0.15) | 0.8872 |
| α - Thalassaemia |  |  |  |  |  |  |  |  |
| Normal | Ref. | . | Ref. | . | Ref. | . | Ref. | . |
| Heterozygous | -0.001 (-0.10, 0.09) | 0.9236 | 0.01 (-0.11, 0.13) | 0.8471 | 0.04 (-0.07, 0.15) | 0.4963 | 0.01 (-0.09, 0.11) | 0.8500 |
| Homozygous | -0.04 (-0.18, 0.09) | 0.5056 | -0.10 (-0.24, 0.03) | 0.1315 | -0.03 (-0.2, 0.14) | 0.7373 | -0.07 (-0.25, 0.11) | 0.4667 |
| Cohort |  |  |  |  |  |  |  |  |
| Junju | Ref. | . | Ref. | . | Ref. | . | Ref. | . |
| Ngerenya-early | -0.12 (-0.28, 0.04) | 0.1352 | -0.02 (-0.23, 0.19) | 0.8785 | -0.11 (-0.27, 0.06) | 0.1978 | -0.11 (-0.26, 0.05) | 0.1739 |
| Ngerenya-late | 0.01 (-0.10, 0.12) | 0.8821 | -0.004 (-0.12, 0.12) | 0.9528 | -0.12 (-0.23, -0.004) | **0.0426** | -0.11 (-0.22, 0.01) | 0.0669 |
| **Covariate** | **AMA1** | | **GE** | |  |  |  |  |
|  | **Estimate**  **(95% CI)** | ***p* value** | **Estimate**  **(95% CI)** | ***p* value** |  |  |  |  |
| Age Group |  |  |  |  |  |  |  |  |
| 0 - 5 years | Ref. | . | Ref. | . |  |  |  |  |
| 6 - 10 years | 0.39 (0.13, 0.64) | **0.0029** | 0.11 (-0.1, 0.32) | **0.3087** |  |  |  |  |
| 11 - 15 years | 0.68 (0.29, 1.07) | **<0.001** | 0.22 (-0.08, 0.53) | **0.1478** |  |  |  |  |
| Asexual parasite positive | 1.04 (0.75, 1.32) | **<0.001** | 0.45 (0.21, 0.68) | **<0.001** |  |  |  |  |
| Gametocyte positive | 0.46 (0.12, 0.81) | **0.0089** | 0.28 (0.02, 0.54) | **0.0313** |  |  |  |  |
| Sickle |  |  |  |  |  |  |  |  |
| Normal | Ref. | . | Ref. | . |  |  |  |  |
| Heterozygous | -0.09 (-0.41, 0.23) | 0.5899 | 0.13 (-0.09, 0.36) | 0.2453 |  |  |  |  |
| α - Thalassaemia |  |  |  |  |  |  |  |  |
| Normal | Ref. | . | Ref. | . |  |  |  |  |
| Heterozygous | -0.13 (-0.4, 0.14) | 0.3335 | -0.24 (-0.45, -0.03) | **0.0222** |  |  |  |  |
| Homozygous | -0.05 (-0.4, 0.31) | 0.7999 | -0.30 (-0.57, -0.04) | **0.0250** |  |  |  |  |
| **Covariate** | **AMA1** | | **GE** | |  |  |  |  |
|  | **Estimate**  **(95% CI)** | ***p* value** | **Estimate**  **(95% CI)** | ***p* value** |  |  |  |  |
| Cohort |  |  |  |  |  |  |  |  |
| Junju | Ref. | . | Ref. | . |  |  |  |  |
| Ngerenya-early | -0.02 (-0.40, 0.36) | 0.9154 | -0.20 (-0.49, 0.10) | **0.1853** |  |  |  |  |
| Ngerenya-late | -0.19 (-0.48, 0.1) | 0.2054 | -0.30 (-0.54, -0.06) | **0.0150** |  |  |  |  |

Ref. – reference category. *P* values in bold are statistically significant (*p* <0.05).

Supplementary Table 7: Univariable linear regression analysis of the factors predicting the magnitude of antibody response to the gametocyte antigens, AMA1 and gametocyte extract – AFIRM cohort

| **Covariate** | **Pfs230** | | **PF3D7_1314500** | | **PF3D7_0303900** | | **PSOP1** | |
| --- | --- | --- | --- | --- | --- | --- | --- | --- |
|  | **Estimate**  **(95% CI)** | ***p* value** | **Estimate**  **(95% CI)** | ***p* value** | **Estimate**  **(95% CI)** | ***p* value** | **Estimate**  **(95% CI)** | ***p* value** |
| Age Group |  |  |  |  |  |  |  |  |
| 0 - 5 years | Ref. | . | Ref. | . | Ref. | . | Ref. | . |
| 6 - 15 years | 0.18 (0.02, 0.33) | **0.0283** | 0.13 (-0.07, 0.33) | 0.2029 | -0.06 (-0.24, 0.12) | 0.4938 | 0.06 (-0.09, 0.22) | 0.3996 |
| > 15 years | 0.58 (0.42, 0.74) | **<0.001** | 0.28 (0.07, 0.48) | **0.0078** | 0.16 (-0.02, 0.34) | 0.0893 | 0.48 (0.32, 0.63) | **<0.001** |
| Parasitaemia |  |  |  |  |  |  |  |  |
| Parasite negative | Ref. | . | Ref. | . | Ref. | . | Ref. | . |
| Sub-patent only | 0.19 (0.03, 0.35) | **0.0182** | 0.35 (0.17, 0.52) | **<0.001** | 0.23 (0.07, 0.38) | **0.0043** | 0.09 (-0.06, 0.23) | 0.2295 |
| Patent | 0.13 (-0.12, 0.38) | 0.3111 | 0.39 (0.13, 0.64) | **0.0029** | 0.45 (0.22, 0.67) | **<0.001** | 0.21 (0, 0.41) | 0.0555 |
| Gametocyte positive | 0.26 (0.09, 0.44) | **0.0035** | 0.32 (0.13, 0.51) | **0.0011** | 0.31 (0.14, 0.47) | **<0.001** | 0.17 (0.02, 0.33) | **0.0293** |
| Sickle |  |  |  |  |  |  |  |  |
| Normal | Ref. | . | Ref. | . | Ref. | **.** | Ref. | . |
| Heterozygous | 0.14 (-0.05, 0.34) | 0.1535 | -0.19 (-0.41, 0.03) | 0.0883 | -0.23 (-0.43, -0.04) | **0.0198** | 0.01 (-0.17, 0.19) | 0.9407 |
| α - Thalassaemia |  |  |  |  |  |  |  |  |
| Normal | Ref. | . | Ref. | . | Ref. | . | Ref. | . |
| Heterozygous | -0.11 (-0.29, 0.06) | 0.2098 | -0.05 (-0.24, 0.14) | 0.5912 | 0.03 (-0.14, 0.20) | 0.7317 | -0.02 (-0.18, 0.13) | 0.7517 |
| Homozygous | -0.1 (-0.32, 0.13) | 0.4180 | -0.11 (-0.36, 0.13) | 0.3688 | 0.12 (-0.09, 0.34) | 0.2631 | 0.10 (-0.09, 0.30) | 0.2999 |
| Season |  |  |  |  |  |  |  |  |
| Dry | Ref. | . | Ref. | . | Ref. | . | Ref. | . |
| Wet | -0.16 (-0.31, -0.01) | **0.0429** | -0.13 (-0.3, 0.04) | 0.1241 | 0.13 (-0.02, 0.28) | 0.0827 | 0.04 (-0.09, 0.18) | 0.5300 |
| **Covariate** | **PF3D7_0208800** | | **MDV1** | | **G377B 3D7** | | **G3D7 PfKE04** | |
|  | **Estimate**  **(95% CI)** | ***p* value** | **Estimate**  **(95% CI)** | ***p* value** | **Estimate**  **(95% CI)** | ***p* value** | **Estimate**  **(95% CI)** | ***p* value** |
| Age Group |  |  |  |  |  |  |  |  |
| 0 - 5 years | Ref. | . | Ref. | . | Ref. | . | Ref. | . |
| 6 - 15 years | 0.02 (-0.13, 0.17) | 0.8341 | 0.16 (-0.01, 0.33) | 0.0593 | 0.16 (-0.0003, 0.33) | 0.0518 | 0.18 (0.003, 0.35) | **0.0468** |
| > 15 years | 0.07 (-0.08, 0.22) | 0.3542 | 0.44 (0.28, 0.61) | **<0.001** | 0.48 (0.31, 0.64) | **<0.001** | 0.51 (0.33, 0.68) | **<0.001** |
| **Covariate** | **PF3D7_0208800** | | **MDV1** | | **G377B 3D7** | | **G3D7 PfKE04** | |
|  | **Estimate**  **(95% CI)** | ***p* value** | **Estimate**  **(95% CI)** | ***p* value** | **Estimate**  **(95% CI)** | ***p* value** | **Estimate**  **(95% CI)** | ***p* value** |
| Parasitaemia |  |  |  |  |  |  |  |  |
| Parasite negative | Ref. | . | Ref. | **.** | Ref. | **.** | Ref. | **.** |
| Sub-patent only | 0.18 (0.05, 0.31) | **0.0086** | 0.37 (0.22, 0.51) | **<0.001** | 0.39 (0.24, 0.54) | **<0.001** | 0.42 (0.27, 0.58) | **<0.001** |
| Patent | 0.26 (0.08, 0.45) | **0.0061** | 0.41 (0.2, 0.63) | **0.0002** | 0.34 (0.13, 0.55) | **0.0017** | 0.40 (0.18, 0.62) | **<0.001** |
| Gametocyte positive | 0.17 (0.03, 0.31) | **0.0162** | 0.32 (0.16, 0.49) | **<0.001** | 0.33 (0.17, 0.49) | **<0.001** | 0.37 (0.20, 0.54) | **<0.001** |
| Sickle |  |  |  |  |  |  |  |  |
| Normal | Ref. | . | Ref. | . | Ref. | . | Ref. | . |
| Heterozygous | -0.05 (-0.21, 0.11) | 0.5581 | -0.03 (-0.22, 0.16) | 0.7558 | -0.08 (-0.27, 0.11) | 0.3939 | -0.17 (-0.37, 0.03) | 0.0947 |
| α - Thalassaemia |  |  |  |  |  |  |  |  |
| Normal | Ref. | . | Ref. | . | Ref. | . | Ref. | . |
| Heterozygous | -0.05 (-0.19, 0.09) | 0.4766 | -0.07 (-0.24, 0.09) | 0.3789 | -0.07 (-0.23, 0.1) | 0.4392 | -0.01 (-0.18, 0.17) | 0.9209 |
| Homozygous | 0.02 (-0.16, 0.2) | 0.8420 | 0.08 (-0.13, 0.29) | 0.4726 | -0.08 (-0.28, 0.13) | 0.4834 | -0.02 (-0.24, 0.21) | 0.8789 |
| Season |  |  |  |  |  |  |  |  |
| Dry | Ref. | . | Ref. | . | Ref. | . | Ref. | . |
| Wet | 0.01 (-0.12, 0.13) | 0.9365 | -0.02 (-0.17, 0.13) | 0.7848 | -0.01 (-0.16, 0.13) | 0.8883 | -0.04 (-0.2, 0.11) | 0.5927 |
| **Covariate** | **AMA1** | | **GE** | |  |  |  |  |
|  | **Estimate**  **(95% CI)** | ***p* value** | **Estimate**  **(95% CI)** | ***p* value** |  |  |  |  |
| Age Group |  |  |  |  |  |  |  |  |
| 0 - 5 years | Ref. | . | Ref. | . |  |  |  |  |
| 6 - 15 years | 0.40 (0.11, 0.69) | **<0.001** | 0.26 (0.002, 0.52) | **0.0498** |  |  |  |  |
| > 15 years | 0.81 (0.52, 1.10) | **<0.001** | 0.83 (0.57, 1.09) | **<0.001** |  |  |  |  |
| Parasitaemia |  |  |  |  |  |  |  |  |
| Parasite negative | Ref. | **.** | Ref. | **.** |  |  |  |  |
| Sub-patent only | 0.45 (0.19, 0.70) | **<0.001** | 0.54 (0.30, 0.77) | **<0.001** |  |  |  |  |
| Patent | 0.91 (0.54, 1.27) | **<0.001** | 0.57 (0.23, 0.91) | **0.0011** |  |  |  |  |
| Gametocyte positive | 0.79 (0.53, 1.06) | **<0.001** | 0.47 (0.22, 0.73) | **<0.001** |  |  |  |  |
| Sickle |  |  |  |  |  |  |  |  |
| Normal | Ref. | . | Ref. | . |  |  |  |  |
| Heterozygous | 0.03 (-0.30, 0.36) | 0.8497 | -0.15 (-0.45, 0.15) | 0.3348 |  |  |  |  |
| **Covariate** | **AMA1** | | **GE** | |  |  |  |  |
|  | **Estimate**  **(95% CI)** | ***p* value** | **Estimate**  **(95% CI)** | ***p* value** |  |  |  |  |
| α - Thalassaemia |  |  |  |  |  |  |  |  |
| Normal | Ref. | . | Ref. | . |  |  |  |  |
| Heterozygous | 0.08 (-0.20, 0.37) | 0.5592 | -0.11 (-0.37, 0.15) | 0.3958 |  |  |  |  |
| Homozygous | 0.26 (-0.10, 0.62) | 0.1582 | 0.12 (-0.21, 0.45) | 0.4614 |  |  |  |  |
| Season |  |  |  |  |  |  |  |  |
| Dry | Ref. | . | Ref. | . |  |  |  |  |
| Wet | -0.05 (-0.30, 0.20) | 0.6924 | -0.04 (-0.27, 0.19) | 0.7427 |  |  |  |  |

Ref. – reference category. *P* values in bold are statistically significant (*p* <0.05).

Supplementary Table 8: Multivariable linear regression models of the factors predicting the magnitude of antibody response to the gametocyte antigens, AMA1 and gametocyte extract – AFIRM cohort

| **Covariate** | **Pfs230** | | **PF3D7_1314500** | | **PF3D7_0303900** | | **PSOP1** | |
| --- | --- | --- | --- | --- | --- | --- | --- | --- |
|  | **Estimate**  **(95% CI)** | ***p* value** | **Estimate**  **(95% CI)** | ***p* value** | **Estimate**  **(95% CI)** | ***p* value** | **Estimate**  **(95% CI)** | ***p* value** |
| Age Group |  |  |  |  |  |  |  |  |
| 0 - 5 years | Ref. | . | Ref. | . | Ref. | . | Ref. | . |
| 6 - 15 years | 0.19 (0.03, 0.34) | 0.0172 | 0.10 (-0.09, 0.3) | 0.3032 | -0.08 (-0.25, 0.09) | 0.3566 | 0.07 (-0.08, 0.22) | 0.3905 |
| > 15 years | 0.56 (0.40, 0.71) | **<0.001** | 0.26 (0.07, 0.46) | **0.0093** | 0.18 (0, 0.35) | **0.0453** | 0.50 (0.35, 0.65) | **<0.001** |
| Parasitaemia |  |  |  |  |  |  |  |  |
| Parasite negative | Ref. | . | Ref. | . | Ref. | . | Ref. | . |
| Sub-patent only | 0.07 (-0.08, 0.22) | 0.3704 | 0.3 (0.11, 0.49) | **0.0028** | 0.19 (0.02, 0.36) | **0.0300** | 0.04 (-0.11, 0.19) | 0.5671 |
| Patent | -0.06 (-0.34, 0.23) | 0.6936 | 0.35 (0.01, 0.68) | **0.0436** | 0.36 (0.07, 0.66) | **0.0169** | 0.20 (-0.06, 0.46) | 0.1309 |
| Gametocyte positive | 0.23 (0.02, 0.44) | **0.0300** | 0.11 (-0.14, 0.37) | 0.3879 | 0.13 (-0.09, 0.35) | 0.2403 | 0.07 (-0.12, 0.26) | 0.4809 |
| Sickle |  |  |  |  |  |  |  |  |
| Normal | Ref. | . | Ref. | . | Ref. | . | Ref. | . |
| Heterozygous | 0.13 (-0.03, 0.30) | 0.1091 | -0.18 (-0.39, 0.03) | 0.0932 | -0.24 (-0.42, -0.05) | **0.0126** | 0.001 (-0.16, 0.16) | 0.9899 |
| α - Thalassaemia |  |  |  |  |  |  |  |  |
| Normal | Ref. | . | Ref. | . | Ref. | . | Ref. | . |
| Heterozygous | -0.08 (-0.24, 0.07) | 0.2736 | 0.03 (-0.15, 0.22) | 0.7362 | 0.12 (-0.04, 0.28) | 0.1567 | -0.01 (-0.16, 0.13) | 0.8635 |
| Homozygous | -0.06 (-0.25, 0.13) | 0.5541 | -0.07 (-0.30, 0.17) | 0.5835 | 0.16 (-0.04, 0.37) | 0.1187 | 0.11 (-0.07, 0.29) | 0.2415 |
| Season |  |  |  |  |  |  |  |  |
| Dry | Ref. | . | Ref. | . | Ref. | . | Ref. | . |
| Wet | -0.11 (-0.24, 0.02) | 0.0982 | -0.12 (-0.28, 0.04) | 0.1512 | 0.11 (-0.03, 0.26) | 0.1189 | 0.06 (-0.06, 0.19) | 0.3429 |
| **Covariate** | **PF3D7_0208800** | | **MDV1** | | **G377B 3D7** | | **G3D7 PfKE04** | |
|  | **Estimate**  **(95% CI)** | ***p* value** | **Estimate**  **(95% CI)** | ***p* value** | **Estimate**  **(95% CI)** | ***p* value** | **Estimate**  **(95% CI)** | ***p* value** |
| Age Group |  |  |  |  |  |  |  |  |
| 0 - 5 years | Ref. | . | Ref. | . | Ref. | . | Ref. | . |
| 6 - 15 years | 0.01 (-0.14, 0.16) | 0.9012 | 0.15 (-0.004, 0.31) | 0.0574 | 0.16 (0.003, 0.31) | **0.0500** | 0.16 (-0.01, 0.32) | **0.0593** |
| > 15 years | 0.08 (-0.07, 0.23) | 0.2978 | 0.45 (0.30, 0.61) | **<0.001** | 0.48 (0.32, 0.63) | **<0.001** | 0.50 (0.34, 0.66) | **<0.001** |
|  |  |  |  |  |  |  |  |  |
|  |  |  |  |  |  |  |  |  |
| **Covariate** | **PF3D7_0208800** | | **MDV1** | | **G377B 3D7** | | **G3D7 PfKE04** | |
|  | **Estimate**  **(95% CI)** | ***p* value** | **Estimate**  **(95% CI)** | ***p* value** | **Estimate**  **(95% CI)** | ***p* value** | **Estimate**  **(95% CI)** | ***p* value** |
| Parasitaemia |  |  |  |  |  |  |  |  |
| Parasite negative | Ref. | . | Ref. | **.** | Ref. | **.** | Ref. | . |
| Sub-patent only | 0.16 (0.01, 0.31) | **0.0387** | 0.32 (0.16, 0.48) | **<0.001** | 0.34 (0.19, 0.49) | **<0.001** | 0.36 (0.2, 0.52) | **<0.001** |
| Patent | 0.25 (-0.01, 0.5) | 0.0633 | 0.40 (0.12, 0.67) | **0.0047** | 0.29 (0.03, 0.56) | **0.0329** | 0.34 (0.06, 0.62) | **0.0175** |
| Gametocyte positive | 0.03 (-0.16, 0.23) | 0.7528 | 0.08 (-0.12, 0.29) | 0.4406 | 0.13 (-0.07, 0.33) | 0.2144 | 0.16 (-0.05, 0.37) | 0.1320 |
| Sickle |  |  |  |  |  |  |  |  |
| Normal | Ref. | . | Ref. | . | Ref. | . | Ref. | . |
| Heterozygous | -0.04 (-0.21, 0.12) | 0.5930 | -0.02 (-0.19, 0.15) | 0.8289 | -0.06 (-0.23, 0.11) | 0.4973 | -0.16 (-0.33, 0.02) | 0.0817 |
| α - Thalassaemia |  |  |  |  |  |  |  |  |
| Normal | Ref. | . | Ref. | . | Ref. | . | Ref. | . |
| Heterozygous | -0.01 (-0.15, 0.14) | 0.9410 | -0.002 (-0.15, 0.15) | 0.9735 | 0.02 (-0.13, 0.16) | 0.8371 | 0.09 (-0.07, 0.24) | 0.2661 |
| Homozygous | 0.03 (-0.15, 0.21) | 0.7257 | 0.09 (-0.09, 0.28) | 0.3253 | -0.05 (-0.24, 0.13) | 0.5879 | 0.02 (-0.17, 0.22) | 0.8111 |
| Season |  |  |  |  |  |  |  |  |
| Dry | Ref. | . | Ref. | . | Ref. | . | Ref. | . |
| Wet | -0.001 (-0.13, 0.12) | 0.9864 | 0.002 (-0.13, 0.13) | 0.9767 | 0.03 (-0.1, 0.16) | 0.6370 | -0.01 (-0.14, 0.13) | 0.9332 |
| **Covariate** | **AMA1** | | **GE** | |  |  |  |  |
|  | **Estimate**  **(95% CI)** | ***p* value** | **Estimate**  **(95% CI)** | ***p* value** |  |  |  |  |
| Age Group |  |  |  |  |  |  |  |  |
| 0 - 5 years | Ref. | . | Ref. | . |  |  |  |  |
| 6 - 15 years | 0.37 (0.11, 0.63) | **0.0065** | 0.25 (0, 0.49) | **0.0472** |  |  |  |  |
| > 15 years | 0.82 (0.55, 1.08) | **<0.001** | 0.85 (0.6, 1.09) | **<0.001** |  |  |  |  |
| Parasitaemia |  |  |  |  |  |  |  |  |
| Parasite negative | Ref. | . | Ref. | . |  |  |  |  |
| Sub-patent only | 0.26 (0, 0.52) | 0.0514 | 0.44 (0.2, 0.68) | **<0.001** |  |  |  |  |
| Patent | 0.56 (0.1, 1.01) | **0.0169** | 0.56 (0.15, 0.98) | **0.0081** |  |  |  |  |
| Gametocyte positive | 0.54 (0.2, 0.88) | **0.0023** | 0.13 (-0.17, 0.44) | 0.3968 |  |  |  |  |
| **Covariate** | **AMA1** | | **GE** | |  |  |  |  |
|  | **Estimate**  **(95% CI)** | ***p* value** | **Estimate**  **(95% CI)** | ***p* value** |  |  |  |  |
| Sickle |  |  |  |  |  |  |  |  |
| Normal | Ref. | . | Ref. | . |  |  |  |  |
| Heterozygous | 0.03 (-0.25, 0.32) | 0.8266 | -0.12 (-0.38, 0.14) | 0.3782 |  |  |  |  |
| α - Thalassaemia |  |  |  |  |  |  |  |  |
| Normal | Ref. | . | Ref. | . |  |  |  |  |
| Heterozygous | 0.23 (-0.02, 0.48) | 0.0770 | -0.03 (-0.26, 0.21) | 0.8213 |  |  |  |  |
| Homozygous | 0.33 (0.02, 0.65) | 0.0380 | 0.16 (-0.13, 0.45) | 0.2711 |  |  |  |  |
| Season |  |  |  |  |  |  |  |  |
| Dry | Ref. | . | Ref. | . |  |  |  |  |
| Wet | -0.02 (-0.24, 0.2) | 0.8789 | -0.02 (-0.22, 0.19) | 0.8835 |  |  |  |  |

Ref. – reference category. *P* values in bold are statistically significant (*p* <0.05).

Supplementary Table 9: Univariable linear regression models of the factors predicting the magnitude of antibody response to the gametocyte antigens, AMA1 and gametocyte extract – AFIRM cohort (patent versus sub-patent analysis)

| **Covariate** | **Pfs230** | | **PF3D7_1314500** | | **PF3D7_0303900** | | **PSOP1** | |
| --- | --- | --- | --- | --- | --- | --- | --- | --- |
|  | **Estimate**  **(95% CI)** | ***p* value** | **Estimate**  **(95% CI)** | ***p* value** | **Estimate**  **(95% CI)** | ***p* value** | **Estimate**  **(95% CI)** | ***p* value** |
| Age Group |  |  |  |  |  |  |  |  |
| 0 - 5 years | Ref. | . | Ref. | . | Ref. | . |  |  |
| 6 - 15 years | 0.06 (-0.17, 0.29) | 0.6045 | 0.02 (-0.20, 0.23) | 0.8791 | -0.31 (-0.58, -0.03) | **0.0300** | -0.01 (-0.22, 0.20) | 0.9287 |
| > 15 years | 0.50 (0.27, 0.73) | **<0.001** | 0.24 (0.02, 0.46) | **0.0312** | -0.03 (-0.30, 0.25) | 0.8466 | 0.39 (0.18, 0.60) | **<0.001** |
| Parasitaemia |  |  |  |  |  |  |  |  |
| Sub-patent | Ref. | . | Ref. | . | Ref. | . | Ref. | . |
| Patent | -0.06 (-0.32, 0.19) | 0.6245 | 0.04 (-0.17, 0.25) | 0.7073 | 0.22 (-0.04, 0.48) | 0.1029 | 0.12 (-0.09, 0.32) | 0.2753 |
| Gametocyte positive | 0.21 (0.003, 0.42) | 0.0505 | 0.10 (-0.08, 0.28) | 0.2643 | 0.16 (-0.07, 0.39) | 0.1696 | 0.13 (-0.05, 0.31) | 0.1685 |
| Sickle |  |  |  |  |  |  |  |  |
| Normal | Ref. | . | Ref. | . | Ref. | . | Ref. | . |
| Heterozygous | 0.09 (-0.21, 0.39) | 0.5616 | -0.12 (-0.37, 0.13) | 0.3417 | -0.19 (-0.50, 0.13) | 0.2429 | 0.02 (-0.23, 0.27) | 0.8916 |
| α - Thalassaemia |  |  |  |  |  |  |  |  |
| Normal | Ref. | . | Ref. | . | Ref. | . | Ref. | . |
| Heterozygous | -0.01 (-0.24, 0.23) | 0.9653 | 0.10 (-0.11, 0.30) | 0.3558 | 0.14 (-0.11, 0.40) | 0.2771 | 0.03 (-0.18, 0.24) | 0.7794 |
| Homozygous | -0.16 (-0.46, 0.14) | 0.2886 | 0.06 (-0.18, 0.31) | 0.5994 | 0.22 (-0.08, 0.52) | 0.1598 | 0.003 (-0.24, 0.25) | 0.9795 |
| Season |  |  |  |  |  |  |  |  |
| Dry | Ref. | . | Ref. | . | Ref. | . | Ref. | . |
| Wet | -0.17 (-0.38, 0.04) | 0.1267 | -0.002 (-0.18, 0.18) | 0.9867 | 0.31 (0.09, 0.53) | **0.0075** | 0.03 (-0.16, 0.21) | 0.7739 |
| **Covariate** | **PF3D7_0208800** | | **MDV1** | | **G377B 3D7** | | **G3D7 PfKE04** | |
|  | **Estimate**  **(95% CI)** | ***p* value** | **Estimate**  **(95% CI)** | ***p* value** | **Estimate**  **(95% CI)** | ***p* value** | **Estimate**  **(95% CI)** | ***p* value** |
| Age Group |  |  |  |  |  |  |  |  |
| 0 - 5 years | Ref. | . | Ref. | . | Ref. | . | Ref. | . |
| 6 - 15 years | -0.15 (-0.35, 0.05) | 0.1370 | 0.07 (-0.15, 0.29) | 0.5219 | 0.04 (-0.15, 0.23) | 0.6993 | 0.1 (-0.13, 0.33) | 0.3918 |
| > 15 years | -0.01 (-0.21, 0.19) | 0.9223 | 0.33 (0.11, 0.56) | **0.0045** | 0.55 (0.36, 0.75) | **<0.001** | 0.62 (0.39, 0.85) | **<0.001** |
|  |  |  |  |  |  |  |  |  |
| **Covariate** | **PF3D7_0208800** | | **MDV1** | | **G377B 3D7** | | **G3D7 PfKE04** | |
|  | **Estimate**  **(95% CI)** | ***p* value** | **Estimate**  **(95% CI)** | ***p* value** | **Estimate**  **(95% CI)** | ***p* value** | **Estimate**  **(95% CI)** | ***p* value** |
| Parasitaemia |  |  |  |  |  |  |  |  |
| Sub-patent | Ref. | . | Ref. | . | Ref. | . | Ref. | . |
| Patent | 0.09 (-0.10, 0.27) | 0.3551 | 0.05 (-0.17, 0.26) | 0.6666 | -0.05 (-0.26, 0.16) | 0.6554 | -0.02 (-0.27, 0.22) | 0.8550 |
| Gametocyte positive | 0.07 (-0.09, 0.24) | 0.3781 | 0.1 (-0.09, 0.29) | 0.2943 | 0.12 (-0.06, 0.30) | 0.1948 | 0.15 (-0.06, 0.36) | 0.1671 |
| Sickle |  |  |  |  |  |  |  |  |
| Normal | Ref. | . | Ref. | . | Ref. | . | Ref. | . |
| Heterozygous | -0.01 (-0.23, 0.22) | 0.9600 | -0.04 (-0.30, 0.22) | 0.7720 | -0.07 (-0.32, 0.18) | 0.5905 | -0.11 (-0.4, 0.18) | 0.4470 |
| α - Thalassaemia |  |  |  |  |  |  |  |  |
| Normal | Ref. | . | Ref. | . | Ref. | . | Ref. | . |
| Heterozygous | 0.05 (-0.13, 0.24) | 0.5845 | 0.03 (-0.18, 0.25) | 0.7588 | 0.10 (-0.11, 0.31) | 0.3561 | 0.16 (-0.08, 0.4) | 0.1893 |
| Homozygous | 0.03 (-0.19, 0.24) | 0.8230 | 0.10 (-0.15, 0.35) | 0.4518 | -0.02 (-0.27, 0.22) | 0.8440 | 0.03 (-0.25, 0.32) | 0.8115 |
| Season |  |  |  |  |  |  |  |  |
| Dry | Ref. | . | Ref. | . | Ref. | . | Ref. | . |
| Wet | 0.03 (-0.13, 0.20) | 0.6873 | 0.07 (-0.12, 0.26) | 0.4536 | -0.02 (-0.21, 0.16) | 0.8260 | 0.01 (-0.2, 0.22) | 0.9320 |
| **Covariate** | **AMA1** | | **GE** | |  |  |  |  |
|  | **Estimate**  **(95% CI)** | ***p* value** | **Estimate**  **(95% CI)** | ***p* value** |  |  |  |  |
| Age Group |  |  |  |  |  |  |  |  |
| 0 - 5 years | Ref. | . | Ref. | . |  |  |  |  |
| 6 - 15 years | 0.16 (-0.23, 0.55) | 0.4234 | 0.19 (-0.11, 0.5) | 0.2189 |  |  |  |  |
| > 15 years | 0.42 (0.03, 0.82) | **0.0373** | 0.61 (0.30, 0.92) | **<0.001** |  |  |  |  |
| Parasitaemia |  |  |  |  |  |  |  |  |
| Sub-patent | Ref. | . | Ref. | . |  |  |  |  |
| Patent | 0.46 (0.10, 0.81) | **0.0137** | 0.04 (-0.27, 0.34) | 0.8093 |  |  |  |  |
| Gametocyte positive | 0.59 (0.28, 0.89) | **<0.001** | 0.15 (-0.12, 0.41) | 0.2846 |  |  |  |  |
| Sickle |  |  |  |  |  |  |  |  |
| Normal | Ref. | . | Ref. | . |  |  |  |  |
| Heterozygous | -0.03 (-0.47, 0.41) | 0.9025 | -0.26 (-0.62, 0.1) | 0.1626 |  |  |  |  |
|  |  |  |  |  |  |  |  |  |
|  |  |  |  |  |  |  |  |  |
| **Covariate** | **AMA1** | | **GE** | |  |  |  |  |
|  | **Estimate**  **(95% CI)** | ***p* value** | **Estimate**  **(95% CI)** | ***p* value** |  |  |  |  |
| α - Thalassaemia |  |  |  |  |  |  |  |  |
| Normal | Ref. | . | Ref. | . |  |  |  |  |
| Heterozygous | 0.31 (-0.05, 0.67) | 0.0935 | 0.07 (-0.24, 0.37) | 0.6604 |  |  |  |  |
| Homozygous | 0.43 (0.01, 0.85) | **0.0453** | 0.16 (-0.20, 0.51) | 0.3870 |  |  |  |  |
| Season |  |  |  |  |  |  |  |  |
| Dry | Ref. | . | Ref. | . |  |  |  |  |
| Wet | 0.12 (-0.20, 0.45) | 0.4528 | 0.05 (-0.22, 0.32) | 0.7099 |  |  |  |  |

Ref. – reference category. *P* values in bold are statistically significant (*p* <0.05).

Supplementary Table 10: Multivariable linear regression models of the factors predicting the magnitude of antibody response to the gametocyte antigens, AMA1 and gametocyte extract – AFIRM cohort (patent versus sub-patent analysis)

| **Covariate** | **Pfs230** | | **PF3D7_1314500** | | **PF3D7_0303900** | | **PSOP1** | |
| --- | --- | --- | --- | --- | --- | --- | --- | --- |
|  | **Estimate**  **(95% CI)** | ***p* value** | **Estimate**  **(95% CI)** | ***p* value** | **Estimate**  **(95% CI)** | ***p* value** | **Estimate**  **(95% CI)** | ***p* value** |
| Age Group |  |  |  |  |  |  |  |  |
| 0 - 5 years | Ref. | . | Ref. | . | Ref. | . | Ref. | . |
| 6 - 15 years | 0.07 (-0.15, 0.29) | 0.5320 | 0.0004 (-0.22, 0.22) | 0.9989 | -0.31 (-0.57, -0.04) | 0.0242 | -0.01 (-0.22, 0.2) | 0.9050 |
| > 15 years | 0.50 (0.27, 0.73) | **<0.001** | 0.23 (0, 0.46) | 0.0523 | -0.005 (-0.28, 0.27) | 0.9720 | 0.42 (0.21, 0.64) | **<0.001** |
| Parasitaemia |  |  |  |  |  |  |  |  |
| Sub-patent | Ref. | . | Ref. | . | Ref. | . | Ref. | . |
| Patent | -0.13 (-0.39, 0.13) | 0.3420 | 0.03 (-0.21, 0.27) | 0.8124 | 0.13 (-0.16, 0.42) | 0.3932 | 0.16 (-0.07, 0.4) | 0.1669 |
| Gametocyte positive | 0.23 (0.01, 0.45) | 0.0409 | 0.11 (-0.1, 0.32) | 0.3060 | 0.14 (-0.11, 0.39) | 0.2664 | 0.06 (-0.14, 0.26) | 0.5616 |
| Sickle |  |  |  |  |  |  |  |  |
| Normal | Ref. | . | Ref. | . | Ref. | . | Ref. | . |
| Heterozygous | 0.10 (-0.16, 0.35) | 0.4632 | -0.13 (-0.38, 0.12) | 0.3102 | -0.24 (-0.54, 0.06) | 0.1200 | 0.02 (-0.21, 0.26) | 0.8559 |
| α - Thalassaemia |  |  |  |  |  |  |  |  |
| Normal | Ref. | . | Ref. | . | Ref. | . | Ref. | . |
| Heterozygous | -0.07 (-0.28, 0.14) | 0.5214 | 0.08 (-0.13, 0.29) | 0.4394 | 0.17 (-0.08, 0.42) | 0.1870 | -0.03 (-0.23, 0.17) | 0.7544 |
| Homozygous | -0.18 (-0.44, 0.08) | 0.1729 | 0.10 (-0.14, 0.34) | 0.4260 | 0.28 (-0.01, 0.57) | 0.0594 | 0.02 (-0.21, 0.25) | 0.8697 |
| Season |  |  |  |  |  |  |  |  |
| Dry | Ref. | . | Ref. | . | Ref. | . | Ref. | . |
| Wet | -0.12 (-0.31, 0.07) | 0.2073 | -0.001 (-0.19, 0.18) | 0.9921 | 0.26 (0.04, 0.48) | **0.0235** | 0.02 (-0.16, 0.19) | 0.8544 |
| **Covariate** | **PF3D7_0208800** | | **MDV1** | | **G377B 3D7** | | **G3D7 PfKE04** | |
|  | **Estimate**  **(95% CI)** | ***p* value** | **Estimate**  **(95% CI)** | ***p* value** | **Estimate**  **(95% CI)** | ***p* value** | **Estimate**  **(95% CI)** | ***p* value** |
| Age Group |  |  |  |  |  |  |  |  |
| 0 - 5 years | Ref. | . | Ref. | . | Ref. | . | Ref. | . |
| 6 - 15 years | -0.16 (-0.36, 0.05) | 0.1349 | 0.07 (-0.16, 0.3) | 0.5435 | 0.03 (-0.17, 0.23) | 0.7662 | 0.09 (-0.14, 0.32) | 0.4525 |
| > 15 years | -0.01 (-0.22, 0.21) | 0.9553 | 0.35 (0.12, 0.59) | **0.0041** | 0.54 (0.33, 0.74) | **<0.001** | 0.6 (0.36, 0.84) | **<0.001** |
| Parasitaemia |  |  |  |  |  |  |  |  |
| Sub-patent | Ref. | . | Ref. | . | Ref. | . | Ref. | . |
| Patent | 0.07 (-0.15, 0.3) | 0.5233 | 0.05 (-0.2, 0.3) | 0.7099 | -0.02 (-0.24, 0.2) | 0.8530 | -0.01 (-0.27, 0.24) | 0.9141 |
| **Covariate** | **PF3D7_0208800** | | **MDV1** | | **G377B 3D7** | | **G3D7 PfKE04** | |
|  | **Estimate**  **(95% CI)** | ***p* value** | **Estimate**  **(95% CI)** | ***p* value** | **Estimate**  **(95% CI)** | ***p* value** | **Estimate**  **(95% CI)** | ***p* value** |
| Parasitaemia |  |  |  |  |  |  |  |  |
| Sub-patent | Ref. | . | Ref. | . | Ref. | . | Ref. | . |
| Patent | 0.07 (-0.15, 0.3) | 0.5233 | 0.05 (-0.2, 0.3) | 0.7099 | -0.02 (-0.24, 0.2) | 0.8530 | -0.01 (-0.27, 0.24) | 0.9141 |
| Gametocyte positive | 0.06 (-0.14, 0.25) | 0.5708 | 0.09 (-0.13, 0.3) | 0.4404 | 0.14 (-0.05, 0.33) | 0.1537 | 0.17 (-0.05, 0.39) | 0.1306 |
| Sickle |  |  |  |  |  |  |  |  |
| Normal | Ref. | . | Ref. | . | Ref. | . | Ref. | . |
| Heterozygous | -0.03 (-0.26, 0.2) | 0.8263 | -0.03 (-0.29, 0.22) | 0.7888 | -0.06 (-0.28, 0.17) | 0.6255 | -0.10 (-0.36, 0.16) | 0.4465 |
| α - Thalassaemia |  |  |  |  |  |  |  |  |
| Normal | Ref. | . | Ref. | . | Ref. | . | Ref. | . |
| Heterozygous | 0.05 (-0.14, 0.25) | 0.5942 | -0.0005 (-0.22, 0.22) | 0.9966 | 0.04 (-0.15, 0.23) | 0.7002 | 0.10 (-0.12, 0.33) | 0.3611 |
| Homozygous | 0.05 (-0.18, 0.27) | 0.6800 | 0.11 (-0.13, 0.36) | 0.3688 | 0.01 (-0.21, 0.23) | 0.9336 | 0.08 (-0.18, 0.33) | 0.5654 |
| Season |  |  |  |  |  |  |  |  |
| Dry | Ref. | . | Ref. | . | Ref. | . | Ref. | . |
| Wet | 0.01 (-0.16, 0.18) | 0.9097 | 0.08 (-0.11, 0.27) | 0.4150 | 0.01 (-0.16, 0.17) | 0.9429 | 0.04 (-0.15, 0.24) | 0.6821 |
| **Covariate** | **AMA1** | | **GE** | |  |  |  |  |
|  | **Estimate**  **(95% CI)** | ***p* value** | **Estimate**  **(95% CI)** | ***p* value** |  |  |  |  |
| Age Group |  |  |  |  |  |  |  |  |
| 0 - 5 years | Ref. | . | Ref. | . |  |  |  |  |
| 6 - 15 years | 0.12 (-0.24, 0.48) | 0.5019 | 0.18 (-0.14, 0.49) | 0.2757 |  |  |  |  |
| > 15 years | 0.43 (0.06, 0.81) | **0.0260** | 0.62 (0.29, 0.94) | **<0.001** |  |  |  |  |
| Parasitaemia |  |  |  |  |  |  |  |  |
| Sub-patent | Ref. | . | Ref. | . |  |  |  |  |
| Patent | 0.21 (-0.18, 0.60) | 0.2959 | 0.07 (-0.27, 0.40) | 0.7079 |  |  |  |  |
| Gametocyte positive | 0.56 (0.23, 0.90) | **0.0015** | 0.14 (-0.15, 0.43) | 0.3498 |  |  |  |  |
| Sickle |  |  |  |  |  |  |  |  |
| Normal | Ref. | . | Ref. | . |  |  |  |  |
| Heterozygous | -0.09 (-0.49, 0.32) | 0.6725 | -0.24 (-0.59, 0.1) | 0.1714 |  |  |  |  |
| **Covariate** | **AMA1** | | **GE** | |  |  |  |  |
|  | **Estimate**  **(95% CI)** | ***p* value** | **Estimate**  **(95% CI)** | ***p* value** |  |  |  |  |
| α - Thalassaemia |  |  |  |  |  |  |  |  |
| Normal | Ref. | . | Ref. | . |  |  |  |  |
| Heterozygous | 0.34 (-0.004, 0.68) | 0.0560 | 0.01 (-0.29, 0.31) | 0.9502 |  |  |  |  |
| Homozygous | 0.54 (0.15, 0.93) | **0.0080** | 0.19 (-0.15, 0.53) | 0.2808 |  |  |  |  |
| Season |  |  |  |  |  |  |  |  |
| Dry | Ref. | . | Ref. | . |  |  |  |  |
| Wet | 0.1 (-0.20, 0.40) | 0.5268 | 0.07 (-0.20, 0.33) | 0.6202 |  |  |  |  |

Ref. – reference category. *P* values in bold are statistically significant (*p <*0.05).

Supplementary Table 11: Primers used for amplification of candidate gametocyte antigens

| **Primer Name** | **Primer Sequence** | **Expression System*** |
| --- | --- | --- |
| APP_WG_Fwd | TAGTTCTCGAGATGTTAAATATTTTTAATAAGGGTAAGAGGC | WGCFS |
| APP_WG_Rev | GCGGCCGCTCAATGGTGGTGATGGTGGTGATTGTTATGAATCGCAATTGGTTCTG | WGCFS |
| PF3D7_1118900_WG_Fwd | TAGTTCTCGAGATGAACTATAACATAAAAACGATAAAATTAATATTTG | WGCFS |
| PF3D7_1118900_WG_Rev | GCGGCCGCTCAATGGTGGTGATGGTGGTGTTTATAGTATATTATTAAATCGTCTATTCCC | WGCFS |
| PF3D7_0513000_WG_Fwd | TAGTTCTCGAGATGGATAATGAATTGTCTGTCAAGGGGAGTTTG | WGCFS |
| PF3D7_0513000_WG_Rev | GCGGCCGCTCAATGGTGGTGATGGTGGTGAATAAGGTATATTTGAAAATTGGATATTCCC | WGCFS |
| PF3D7_1105800_WG_Fwd | TAGTTGGTACCATGTTAACATATATGTTAATGAAAGACGAAG | WGCFS |
| PF3D7_1105800_WG_Rev | GCGGCCGCTCAATGGTGGTGATGGTGGTGATATGCTCCTTCTTTGTCGCAC | WGCFS |
| PF3D7_0208800_WG_Rev | GCGGCCGCTCAATGGTGGTGATGGTGGTGTGATGTGTTATCTGGTGGCTGGGG | WGCFS |
| PF3D7_0309100_WG_Fwd | TAGTTCTCGAGATGGATGTAATAAATAAGACACAAGAAAATTATTTAG | WGCFS |
| PF3D7_0309100_WG_Rev | GCGGCCGCTCAATGGTGGTGATGGTGGTGTAGCTCCTCATTTTTTATGAACACCC | WGCFS |
| PF3D7_1251000_WG_Fwd | TAGTTCTCGAGATGAATGTAGATAAAGAAGTGCCAACAGTTAAATGGGG | WGCFS |
| PF3D7_1251000_WG_Rev | GCGGCCGCTCAATGGTGGTGATGGTGGTGATATTCGTGTAATGAGAAGTTTGG | WGCFS |
| PF3D7_1314500_WG_rev | GCGGCCGCTCAATGGTGGTGATGGTGGTGAACTATAGTTTTTATTTCAAAAAAAG | WGCFS |
| G377_WG_A2_Fwd | TAGTTCTCGAGATGCCTAACGTTGTGGAATTAACTCCTGAAGAG | WGCFS |
| G377_WG_A2_Rev | GCGGCCGCTCAATGGTGGTGATGGTGGTGTTTAAAATCAAAAGCATGTACCTCATATATTTG | WGCFS |
| G377_WG_B_Fwd | TAGTTCTCGAGATGCCTGAACCATGGCCTCTTGATGAATCGCC | WGCFS |
| G377_WG_B_Rev | GCGGCCGCTCAATGGTGGTGATGGTGGTGATTTGTTTTTGTTTGGCTAGTCAAATCATC | WGCFS |
| GECO/GEXP01_3D7_Fwd | TAGTTCTCGAGATGTTTGTATATAATAATGTAGGAAGAAAACG | WGCFS |
| GECO/GEXP01_3D7_Rev | GCGGCCGCTCAATGGTGGTGATGGTGGTGAACATTGATCTTTTGGAGTATCATACTTCC | WGCFS |
| GECO/GEXP01_PfKE04_Fwd | TAGTTCTCGAGATGTTTGTATATAATAATGTAGGAAGAAAACG | WGCFS |
| GECO/GEXP01_PfKE04_Rev | GGCGGCCGCTCAATGGTGGTGATGGTGGTGAACATTAATCTTTTGGAGTATCATACTTCC | WGCFS |
| LAP5_WG_Fwd | TAGTTCTCGAGATGCAAAATTATGATAAGGAAAATTTAAAGAAACTG | WGCFS |
| LAP5_WG_Rev | GCGGCCGCTCAATGGTGGTGATGGTGGTGGTTATGAAGGAATAATATCTGTATAGATTG | WGCFS |
| MDV1_WG_Rev | GCGGCCGCTCAATGGTGGTGATGGTGGTGATCACTATCACTGTGTGTTTTTATATCC | WGCFS |
| MVD1_WG_Fwd | TAGTTCTCGAGATGGATATTTATTACCATTGTATTAACATAGG | WGCFS |
| NOT1 Domain 1_WG_Rev | GCGGCCGCTCAATGGTGGTGATGGTGGTGTTTTGATTGTAAACAACTTTTTGTGATG | WGCFS |
| NOT1 Domain 1_WG_Fwd | TAGTTCTCGAGATGCTCTTCAAATATTAACGATAATATTATTTT | WGCFS |
| NOT1 Domain 2_WG_Fwd | TAGTTCTCGAGATGTACTAATAATTTTAACCTACCTGTGG | WGCFS |
| NOT1 Domain 2_Rev | GCGGCCGCTCAATGGTGGTGATGGTGGTGCATTATTTAATATATTACAAATATTG | WGCFS |
| P47_WG_Fwd | TAGTTCTCGAGATGGAACTATTAAGCTCAACACAATACG | WGCFS |
| P47_WG_Rev | GCGGCCGCTCAATGGTGGTGATGGTGGTGAGATGCGATATGTAATTCCATTACTGC | WGCFS |
| PF3D7_0303900_WG_Rev | GCGGCCGCTCAATGGTGGTGATGGTGGTGTATGTGTATTTTTTTTTTGGGTATACATAATG | WGCFS |
| PF3D7_0208800_WG_Fwd | TAGTTCTCGAGATGAAACAAACTATTCTTAAGTTATCATATC | WGCFS |
| PF3D7_0303900_WG_Fwd | TAGTTCTCGAGATGGATATTAAGTTGGTAAATTCAGATTTTGG | WGCFS |
| PF3D7_1314500_WG_fwd | TAGTTCTCGAGATGACACATTTTAATATAGGACCATATGAAAAAG | WGCFS |
| PF3D7_1354400_WG_Fwd | TAGTTCTCGAGATGATATTTATATGTGGTACAAGTATTGTTGG | WGCFS |
| Pfs230_WG_Fwd | TAGTTCTCGAGATGGAATATGTAGATGAAAAAGAAAGGCAAGG | WGCFS |
| Pfs230_WG_Rev | GCGGCCGCTCAATGGTGGTGATGGTGGTGCTTAGATATTAATAATTCAACAATTCCG | WGCFS |
| Pfs48/45_WG_Fwd | TAGTTCTCGAGATGAACAATGATTTTTGTAAGCCTAGC | WGCFS |
| Pfs48/45_WG_Rev | GCGGCCGCTCAATGGTGGTGATGGTGGTGATCTATAGTAACTGTCATATAAGC | WGCFS |
| PHISTa_WG_Fwd | TAGTTCTCGAGATGATATTGTGTATGGGAATATTATATTTACC | WGCFS |
| PHISTa_WG_Rev | GCGGCCGCTCAATGGTGGTGATGGTGGTGTTTTTTATAATTTTTTTTTTTTCTTGAAG | WGCFS |
| PIESP15_PfKE04_Fwd | TAGTTCTCGAGATGAAAAATAATAGTTATAATGATTATTACAATAC | WGCFS |
| PIESP15_PfKE04_Rev | GCGGCCGCTCAATGGTGGTGATGGTGGTGTAGGATGTGTTTTTTTTCGACATCTCG | WGCFS |
| PSOP1_WG_Fwd | TAGTTCTCGAGATGGTAAGACCCTACAAGGAACGTTATGG | WGCFS |
| PSOP1_WG_Rev | GCGGCCGCTCAATGGTGGTGATGGTGGTGTGGACTCCTTACAAATGAGGATAAGTTG | WGCFS |
| PSOP12_WG_Fwd | TAGTTCTCGAGATGTTTTATTTTGAACAGACTGAAGAATTAC | WGCFS |
| PSOP12_WG_Rev | GCGGCCGCTCAATGGTGGTGATGGTGGTGTATTAAATTAAATGTAATATCATAGTAC | WGCFS |
| PSOP25_WG_Fwd | TAGTTCTCGAGATGAAAGATGATGGCCAACGTCACGG | WGCFS |
| PSOP25_WG_Rev | GCGGCCGCTCAATGGTGGTGATGGTGGTGTTTACATTTTTCGATATCTTTTTTAATC | WGCFS |
| TLP_WG_Fwd | TAGTTCTCGAGATGGAGAAATTAATTTCTAAATTAACAAAAAATGG | WGCFS |
| TLP_WG_Rev | GCGGCCGCTCAATGGTGGTGATGGTGGTGTTTCCACTGCGTGTCATTCATTATTTC | WGCFS |
| PF3D7_1354400_WG_Rev | GCGGCCGCTCAATGGTGGTGATGGTGGTGTGTTAGTTGAATATCTCCAATTGATACAATAGCG | WGCFS |
| WG Flank-to-Flank Fwd | TCCACTAACCACCTATCTACATCACC | WGCFS |
| WG Flank-to-FLank Rev | CGCTAGCCGTAAATTCTATACAAAACC | WGCFS |
| APP_pGS_Fwd | gcgtagctgaaaccggcCTGAACATCTTCAACAAGGGC | HEK293E |
| APP_pGS_Rev | ggtggctccagctagcGTTGTTGTGGATGGCGATAGG | HEK293E |
| PF3D7_1118900_pGS_Fwd | gcgtagctgaaaccggcAACTACAACATCAAGACCATC | HEK293E |
| PF3D7_1118900_pGS_Rev | ggtggctccagctagcTTTGTAGTAGATGATCAGATCG | HEK293E |
| PF3D7_0513000_pGS_Fwd | gcgtagctgaaaccggcGATAACGAGCTGAGCGTGAAGG | HEK293E |
| PF3D7_0513000_pGS_Rev | ggtggctccagctagcGATCAGGTAGATCTGGAAGTTGG | HEK293E |
| PF3D7_1251000_pGS_Fwd | gcgtagctgaaaccggcAATGTGGACAAAGAGGTGCC | HEK293E |
| PF3D7_1251000_pGS_Rev | ggtggctccagctagcGTACTCGTGCAGGCTGAAGTTG | HEK293E |
| G377B_pGS_Fwd | gcgtagctgaaaccggcCCCGAGCCTTGGCCTCTG | HEK293E |
| G377B_pGS_Rev | ggtggctccagctagcGTTGGTCTTGGTCTGGCTGG | HEK293E |
| GEXP01_pGS_Fwd | gcgtagctgaaaccggcTTCGTGTACAACAACGTGGGCAG | HEK293E |
| GEXP01_pGS_Rev | ggtggctccagctagcGACGTTGATCTTCTGCAGGATC | HEK293E |
| NOT1_pGS_Fwd | gcgtagctgaaaccggcACCAACAACTTCAACCTGCC | HEK293E |
| NOT1_pGS_Rev | ggtggctccagctagcCTGGTTGTAGACCACCTTCTGG | HEK293E |
| P47_pGS_Fwd | gcgtagctgaaaccggcGAGCTGCTGAGCAGCACCCAG | HEK293E |
| P47_pGS_Rev | ggtggctccagctagcGCTGGCAATGTGCAGTTCCATC | HEK293E |
| PF3D7_0208800_pGS_Fwd | gcgtagctgaaaccggcAAGCAGACCATCCTGAAGCTG | HEK293E |
| PF3D7_0208800_pGS_Rev | ggtggctccagctagcGCTGGTATTGTCAGGAGGC | HEK293E |
| PF3D7_0303900_pGS_Fwd | gcgtagctgaaaccggcGACATCAAGCTGGTCAACAGCG | HEK293E |
| PF3D7_0303900_pGS_Rev | ggtggctccagctagcGATGTGGATCTTCTTCTTGGGG | HEK293E |
| PF3D7_1314500_pGS_Fwd | gcgtagctgaaaccggcACCCACTTCAACATCGGCCC | HEK293E |
| PF3D7_1314500_pGS_Rev | ggtggctccagctagcTTTGCTGTTCATCTTCTCATTGAAC | HEK293E |
| PHISTa_pGS_Fwd | gcgtagctgaaaccggcATGAAGAACACCGGCAGCAAC | HEK293E |
| PHISTa_pGS_Rev | ggtggctccagctagcTTTCTTGTAGTTCTTCTTCTTCCGG | HEK293E |
| PIESP15_pGS_Fwd | gcgtagctgaaaccggcAAGAACAACAGCTACAACGAC | HEK293E |
| PIESP15_pGS_Rev | ggtggctccagctagcGTAGTTGCCCTTGTTGATGATC | HEK293E |
| PSOP1_pGS_Fwd | gcgtagctgaaaccggcGTGCGGCCCTACAAAGAGAG | HEK293E |
| PSOP1_pGS_Rev | ggtggctccagctagcAGGGCTCCGCACGAAGCTG | HEK293E |
| PSOP12_pGS_Fwd | gcgtagctgaaaccggcTTCTACTTCGAGCAGACCGAG | HEK293E |
| PSOP12_pGS_Rev | ggtggctccagctagcGATCAGATTGAAGGTGATATCG | HEK293E |

*WGCFS – wheat germ-cell-free expression system. HEK293E – mammalian expression system.
